# Host transcriptomic plasticity and photosymbiotic fidelity underpin *Pocillopora* acclimatization across thermal regimes in the Pacific Ocean

**DOI:** 10.1038/s41467-023-38610-6

**Published:** 2023-06-01

**Authors:** Eric J. Armstrong, Julie Lê-Hoang, Quentin Carradec, Jean-Marc Aury, Benjamin Noel, Benjamin C. C. Hume, Christian R. Voolstra, Julie Poulain, Caroline Belser, David A. Paz-García, Corinne Cruaud, Karine Labadie, Corinne Da Silva, Clémentine Moulin, Emilie Boissin, Guillaume Bourdin, Guillaume Iwankow, Sarah Romac, Sylvain Agostini, Bernard Banaigs, Emmanuel Boss, Chris Bowler, Colomban de Vargas, Eric Douville, Michel Flores, Didier Forcioli, Paola Furla, Pierre E. Galand, Eric Gilson, Fabien Lombard, Stéphane Pesant, Stéphanie Reynaud, Matthew B. Sullivan, Shinichi Sunagawa, Olivier P. Thomas, Romain Troublé, Rebecca Vega Thurber, Didier Zoccola, Serge Planes, Denis Allemand, Patrick Wincker

**Affiliations:** 1 Génomique Métabolique, Genoscope, Institut François Jacob, CEA, CNRS, Univ Evry, Université Paris-Saclay, 91057 Evry, France; 2 Research Federation for the study of Global Ocean Systems Ecology and Evolution, FR2022/ Tara Oceans-GOSEE, 3 rue Michel-Ange, 75016 Paris, France; 3 PSL Université Paris: EPHE-UPVD-CNRS, UAR 3278 CRIOBE, Université de Perpignan, 52 Avenue Paul Alduy, 66860 Perpignan Cedex, France; 4 Department of Biology, University of Konstanz, 78457 Konstanz, Germany; 5 Centro de Investigaciones Biológicas del Noroeste (CIBNOR), Av. IPN 195, La Paz, Baja California Sur 23096 México; 6 Genoscope, Institut François Jacob, CEA, Université Paris-Saclay, Evry, France; 7 Fondation Tara Océan, Base Tara, 8 rue de Prague, 75 012 Paris, France; 8 School of Marine Sciences, University of Maine, Orono, 04469 ME USA; 9 Sorbonne Université, CNRS, Station Biologique de Roscoff, AD2M, UMR 7144, ECOMAP, 29680 Roscoff, France; 10 Shimoda Marine Research Center, University of Tsukuba, 5−10-1, Shimoda, Shizuoka, Japan; 11 Ecole Normale Supérieure, PSL Research University, Institut de Biologie de l’Ecole Normale Supérieure (IBENS), CNRS UMR 8197, INSERM U1024, 46 rue d’Ulm, F-75005 Paris, France; 12 Laboratoire des Sciences du Climat et de l’Environnement, LSCE/IPSL, CEA-CNRS-UVSQ, Université Paris-Saclay, F-91191 Gif-sur-Yvette, France; 13 Weizmann Institute of Science, Department of Earth and Planetary Sciences, 76100 Rehovot, Israel; 14 Université Côte d’Azur, CNRS, INSERM, IRCAN, Medical School, Nice, France; 15 LIA ROPSE, Laboratoire International Associé Université Côte d’Azur - Centre Scientifique de Monaco, Principality of Monaco, Monaco; 16 Sorbonne Université, CNRS, Laboratoire d’Ecogéochimie des Environnements Benthiques (LECOB), Observatoire Océanologique de Banyuls, 66650 Banyuls sur mer, France; 17 Department of Medical Genetics, CHU, Nice, France; 18 Sorbonne Université, Institut de la Mer de Villefranche sur mer, Laboratoire d’Océanographie de Villefranche, F-06230 Villefranche-sur-Mer, France; 19 European Molecular Biology Laboratory, European Bioinformatics Institute, Wellcome Genome Campus, Hinxton, Cambridge, CB10 1SD UK; 20 Centre Scientifique de Monaco, 8 Quai Antoine Ier, MC-98000 Principality of Monaco, Monaco; 21 Departments of Microbiology and Civil, Environmental and Geodetic Engineering, Ohio State University, Columbus, OH 43210 USA; 22 Institute of Microbiology, Department of Biology, Vladimir-Prelog-Weg 4, 8093 Zürich, Switzerland; 23 School of Biological and Chemical Sciences, Ryan institute, University of Galway, University Road H91TK33, Galway, Ireland; 24 Oregon State University, Department of Microbiology, 220 Nash Hall, 97331 Corvallis, OR USA; 25 PSL Research University: EPHE-UPVD-CNRS, USR 3278 CRIOBE, Université de Perpignan, 66860 Perpignan Cedex, France

**Keywords:** Ecophysiology, Marine biology, Gene expression

## Abstract

Heat waves are causing declines in coral reefs globally. Coral thermal responses depend on multiple, interacting drivers, such as past thermal exposure, endosymbiont community composition, and host genotype. This makes the understanding of their relative roles in adaptive and/or plastic responses crucial for anticipating impacts of future warming. Here, we extracted DNA and RNA from 102 *Pocillopora* colonies collected from 32 sites on 11 islands across the Pacific Ocean to characterize host-photosymbiont fidelity and to investigate patterns of gene expression across a historical thermal gradient. We report high host-photosymbiont fidelity and show that coral and microalgal gene expression respond to different drivers. Differences in photosymbiotic association had only weak impacts on host gene expression, which was more strongly correlated with the historical thermal environment, whereas, photosymbiont gene expression was largely determined by microalgal lineage. Overall, our results reveal a three-tiered strategy of thermal acclimatization in *Pocillopora* underpinned by host-photosymbiont specificity, host transcriptomic plasticity, and differential photosymbiotic association under extreme warming.

## Abstract (web summary)

Using data from the Tara Pacific expedition to investigate symbiont fidelity and patterns of gene expression across a thermal gradient, this study shows that *Pocillopora* corals have a three-tiered strategy of thermal acclimatization that is underpinned by host–photosymbiont specificity, host transcriptomic plasticity, and differential photosymbiotic associations under extreme warming.

## Introduction

Coral reefs are ecologically and economically important ecosystems whose existence depends upon the mutualistic, photosymbiotic, association between certain Cnidarian hosts and their dinoflagellate symbionts. Breakdown of this association can lead to expulsion of algal symbionts (i.e., bleaching) and ultimately result in coral mortality and reef loss^1–3^. Recent environmental perturbations, particularly rising sea surface temperatures and increasing frequency of extreme heating events (i.e., marine heatwaves), are disrupting coral-dinoflagellate photosymbioses worldwide, increasing the frequency and severity of coral bleaching events^3^. There is therefore growing concern about the potential for heating-driven local extirpation of coral species and severe reef loss in the current century. However, in some regions, corals exhibit higher than average heat tolerances^4^ or show the ability to rapidly respond to and recover from acute thermal challenges^5^. Repeated sublethal exposure to elevated sea surface temperatures can also drive positive acclimatization (phenotypic plasticity) and/or selection for thermotolerant genotypes (i.e., local adaptation). However, in other species, warm pre-conditioning had no or negative effects on holobiont performance during subsequent thermal challenges^6,7^. The capacity for thermal acclimatization and/or adaptation may therefore play an important role in determining potential winners and losers among coral species in the face of global change^5,8^. Determining whether acclimatization capacity can keep pace with projected environmental change and in which symbiotic assemblages, is therefore of paramount importance for predicting responses of the coral-dinoflagellate photosymbiosis under a warming climate.

Understanding how corals will respond to ongoing climate change requires prior knowledge regarding their capacities for thermal acclimatization (e.g., holobiont flexibility and transcriptomic plasticity) and adaptation^9–11^. The mechanisms underlying these capacities as well as their limits and extent among different coral holobiotypes and the relative roles of each symbiotic partner remain under intense investigation. In some cases, alteration of the dominant photosymbiont lineage, from thermally sensitive to thermally tolerant Symbiodiniaceae for example, confers a higher resistance of the coral holobiont to heat stress^12,13^. In other cases, thermal acclimatization is achieved through altered gene expression in the host and/or symbiont, including high basal expression (i.e., gene frontloading) of certain stress response genes, resulting in increased holobiont thermal tolerance^5,11,14,15^. Coral holobiont thermal sensitivity is therefore dependent on an array of interacting drivers, including environmental history^5,16–19^, endosymbiont community composition^20–23^, and host genotype^15,23–25^. However, the relative role of each of these factors in determining holobiont expression in natura remains poorly resolved. A remaining challenge, therefore, is to better understand how environmental context affects the complex interplay between the host and symbiont and to what extent certain genotypes and or host/symbiont pairings might confer resilience or resistance to projected environmental change. Finally, acclimatization or adaptation of a coral holobiont at the local (reef) scale is often not representative of capacities at larger (ocean) scales and therefore does not allow for accurate global projections of the survival or decline of a species or of reef ecosystems^15^.

Given the variety of components involved in maintaining the coral-dinoflagellate mutualism against environmental perturbation, recent analyses have begun to adopt a more integrative and multidisciplinary approach to understanding the impacts of climate change on coral health. Integration of various analysis methods (genomic, transcriptomic, barcoding, imaging, etc.) is needed to draw a global picture of coral holobiont adaptation and acclimatization capacities^5,15^. The *Tara* Pacific expedition provides a unique opportunity to address these important questions because it allows for comparative analyses of differences in holobiont community composition and photosymbiotic regulation among environments and species across the tropical Pacific Ocean^26^.

**Fig. 1:**
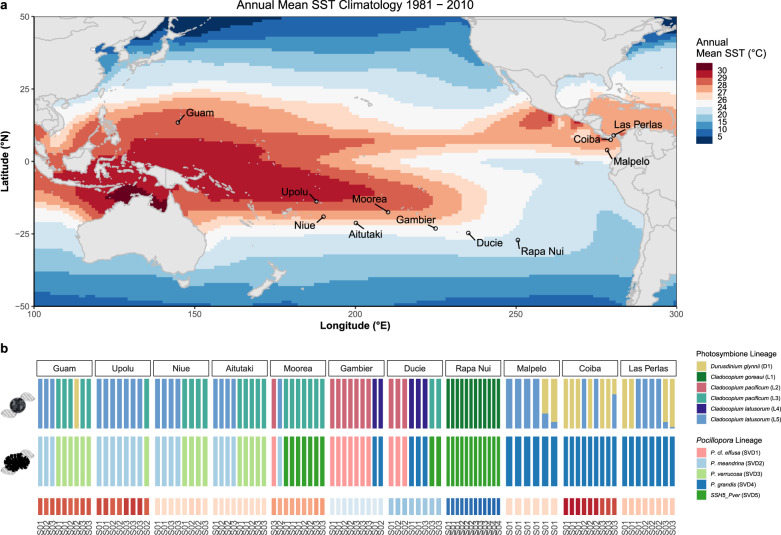
*Pocillopora* and Symbiodiniaceae lineages identified in each sample across the Pacific Ocean. **a** Map showing the 11 islands sampled during the *Tara* Pacific expedition alongside annual mean sea surface temperature (SST) climatologies over the period 1981–2010 (NOAA OI SST V2 High-Resolution SST data). **b** Lineage assignments of sampled colonies for both the photosymbiont (Symbiodiniaceae icon, top panel; *Cladocopium* genetic lineages and *Durusdinium glynnii* when also present) and *Pocillopora* host (Coral colony icon, middle panel). Lineages were identified from an analysis of single nucleotide polymorphisms (SNPs) performed using metagenomic reads for the *Pocillopora* host and on metatranscriptomic reads for the Symbiodiniaceae. In colonies containing both *Durusdinium* and *Cladocopium*, the latter were assigned to *C. latusorum* L5 using the ITS2 pairwise distance clustering (Supplementary Fig. 3). Also displayed are historical mean sea surface temperatures (2002—sampling date) at each reef site (bottom panel, S01/S02/S03—sites 01, 02, and 03). Source data are available for this figure^108^.

To this end, we investigated gene expression profiles of 102 colonies of *Pocillopora* spp. and their associated photosymbionts alongside metagenomic sequences and environmental context data from 11 islands across the Pacific Ocean (Fig. 1a) in order to assess the relative contributions of environmental and genetic factors in determining coral holobiont gene expression in natura. We used dual RNA-seq expression profiling, and variation partitioning to examine the effects of a historical sea surface temperature gradient on transcriptomic plasticity among various *Pocillopora* hosts and their associated dinoflagellate endosymbionts (*Symbiodiniaceae*) across the Pacific Ocean.

Here we show that multiple *Pocillopora* lineages exhibit a high degree of host–photosymbiont fidelity across environments with instances of symbiotic flexibility and/or breakdown associated with corals living on reefs which have historically experienced elevated sea surface temperatures (SSTs). We also describe how host and photosymbiont expression profiles respond to different drivers and discuss how these mechanisms may represent a three-tiered strategy of thermal acclimatization/adaptation in *Pocillopora* corals. Finally, we discuss the implications of this strategy for continued persistence of *Pocillopora* holobionts under current and projected future ocean warming.

## Results

### *Pocillopora* and Symbiodiniaceae identification

*Pocillopora* hosts were taxonomically identified using genome-wide SNPs clustering and coalescent (SVD quartet) analysis as described in ref. ^27^. They obtained five distinct genetic lineages (SVD1 to SVD5), which were further assigned to the species level based on a set of *Pocillopora* reference sequences from refs. ^27,28^ and the mtORF sequences from ref. ^29^. This resulted in the identification of 12 colonies as belonging to *P*. cf. *effusa* (SVD1, GSH01, haplotypes 2 and 11), 19 colonies to *P. meandrina* (SVD2, GSH09b, haplotypes 1a and 8a), 17 colonies to *P. verrucosa* (SVD3, GSH13c, haplotypes 3a,b,f,h), 33 colonies to *P. grandis* (SVD4, GSH09ctsp, haplotype 1a) and 21 colonies to a cryptic *P. verrucosa* species (SVD5, GSH14, haplotype 10), hereafter referred to as SSH5_pver (Fig. 1b). We also identified one hybrid colony *P. meandrina/P. verrucosa* (SVD2/SVD3). The mtORF phylogeny and *Pocillopora* species name connections with previously published studies are reported in Supplementary Fig. 1. Although several studies corroborate these species assignments based on various genomic data^28–30^, complementary analyses of skeletal morphology will be necessary to confirm these species names with the respective type specimen vouchers^31^. We used two different genetic markers (nuclear ITS2 sequences and plastid-encoded psbA^ncr^ sequences) alongside transcriptome-wide SNPs to identify Symbiodiniaceae lineages in each *Pocillopora* colony. The ITS2 sequence profiles revealed that 84 *Pocillopora* colonies hosted *Cladocopium C1/C42-*based profiles, 9 colonies hosted *Durusdinium D1*-based profiles, and 9 colonies contained both *Cladocopium* and *Durusdinium* in varying proportions (Supplementary Fig. 2). For *Durusdinium*-containing colonies, the majority ITS2 sequences were D1 for 15 colonies with D1/D1as, D1/D2d, and D1/D1aa accounting for the 3 remaining colonies (one representative each). These ITS2 profiles are all associated with *Durusdinium glynnii*. To identify the different *Cladocopium* lineages, we aligned metatranscriptomic reads of *Pocillopora* colonies on the predicted genes of the *C. goreaui* genome^32^ and called the SNPs for each *Pocillopora* colony hosting only *Cladocopium*. We clustered the frequencies of 3712 biallelic SNPs distributed across 1354 transcripts and obtained an optimal number of five different clusters potentially corresponding to five different lineages hereafter named L1 to L5 (Fig. 1b and Supplementary Fig. 3a). We then compared the SNP clustering with the clustering of ITS2 profile distances which also include samples containing both *Cladocopium* and *Durusdinium* symbionts (Supplementary Fig. 3b). The topologies of these two dendrograms are highly similar and this clustering reveals that only *Cladocopium* L5 is present in *Pocillopora* colonies hosting both *Cladocopium* and *Durusdinium*. Finally to link each *Cladocopium* lineage to formally described species, we created a phylogeny based on the mapping of metagenomic reads on the non-coding region of the psbA gene (psbA^ncr^) recently sequenced in three *Cladocopium* species: *C. goreaui*, *C. latusorum*, and *C. pacificum*^29,33^. The psbA^ncr^ sequence robustly separated *Cladocopium* L1 from L2/L3 and L4/L5. *Cladocopium* L1 is most similar to *C. goreaui*, whereas L2/L3 are two different taxa within *C. latusorum* and L4/L5 are two different taxa within *C. pacificum* (Supplementary Fig. 3c). Notably, the positioning of two *Cladocopium* samples (I05S03C001 and I05S03C006) was incoherent between the three methods. They were assigned to L3, L2, or L5 according to the SNP, ITS2 and psbA^ncr^ analyses, respectively. Given that the SNP analysis is based on a larger number of variants distributed across the entire transcriptome, in comparison to the short and multi-copy (ITS2) or plastid-encoded (psbA^ncr^) marker sequences, we consider that these two *Cladocopium* populations belong to L3.

### Host–symbiont associations and holobiont biogeography

Three *Pocillopora* species that we sampled (*P*. cf. *effusa*, *P. meandrina* and *P. verrucosa*) exhibited near perfect symbiotic fidelity—i.e., a one to one pairing of photosymbiont lineage and host—which persisted across all sampled environments (Fig. 1b). *P*. cf. *effusa* colonies (present on Ducie, Gambier, and Moorea) were always found in symbiosis with *C. pacificum* (L2). Similarly, *P. meandrina* colonies (present on several islands in the Western Pacific) always hosted *C. latusorum* (L5). Finally, *P. verrucosa* which was present in both the Central and Western Pacific, displayed near perfect symbiotic fidelity with *C. pacificum* (L3) across its range, with only one instance of a difference in photosymbiont association (one colony in Guam hosting *D. glynnii*) which we discuss, below. The *P. meandrina/P. verrucosa* hybrid on Niue island (I09S03C010) hosted *C. pacificum* (L3).

**Fig. 2:**
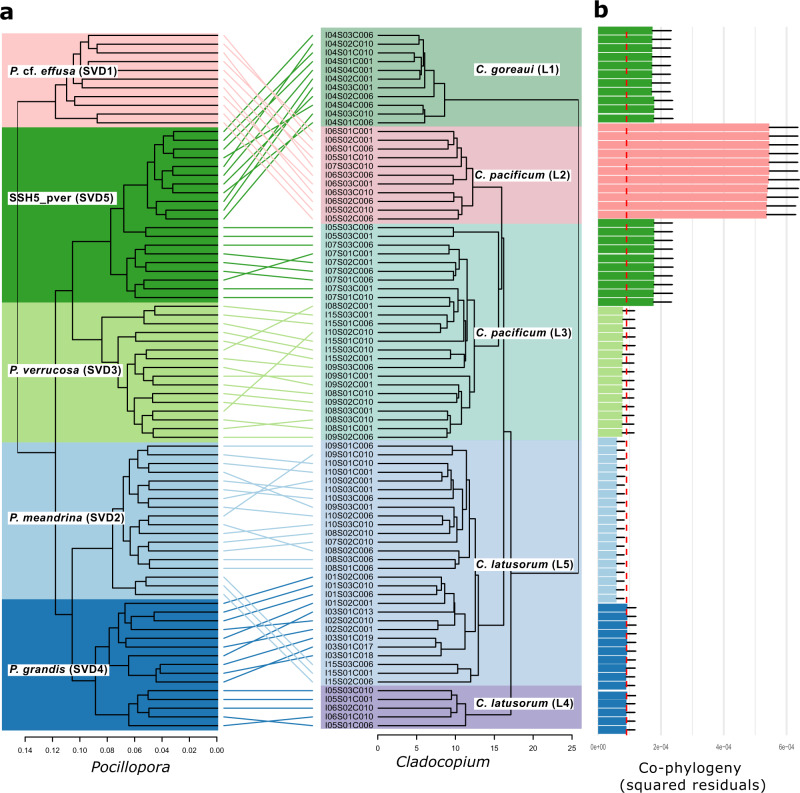
*Pocillopora* and *Cladocopium* cophylogeny. **a** Cophylogeny of *Pocillopora* and its *Cladocopium* photosymbiont. The *Pocillopora* dendrogram (left) is obtained from the maximum likelihood phylogenetic tree of ref. ^27^. The *Cladocopium* dendrogram (right) is obtained from the hierarchical clustering of the frequency of 3712 SNPs detected on the metatranscriptomic reads aligned on 1354 genes of *C. goreaui*. Edge colors correspond to host species assignments. **b** Contributions of individual host–photosymbiont links to the Procrustean fit with jackknifed squared residuals resulting from PACo applied to the patristic distance matrices generated from the *n* = 81 biologically independent samples (colonies hosting *Durusdinium* and the hybrid colony were excluded). Host lineage is indicated by the fill color and bars display sample means and standard deviations (whiskers). The median mean squared residual value is shown in red (dashed line). Lower squared residual values indicate a stronger contribution to host–photosymbiont cophylogeny. Source data for the phylogenies in this figure are available^108^. Source data for the Procrustean analysis are provided as a Source Data file.

The two other *Pocillopora* species (*P. grandis* and SSH5_pver) suggest symbiont flexibility. *Pocillopora SSH5_pver* was the only lineage sampled on Rapa Nui and is in symbiosis with *C. goreaui*. SSH5_pver was also present on Ducie and Moorea islands in symbiosis with *C. pacificum* (L3). The two symbionts of SSH5_pver perfectly match to two genetic subclades of SSH5_pver (Fig. 2a) that are geographically separated. *P. grandis* was the sole lineage present among colonies sampled from the far Eastern Tropical Pacific (Isla de Las Perlas, Coiba, and Malpelo) and hosted *C. latusorum* (L5) and/or *D. glynnii*. *P. grandis* was also present on Ducie and Gambier islands where it was found in association with *C. latusorum* (L4). Similarly, the two *Cladocopium* symbionts of *P. grandis* match two genetic subclades that are geographically separated.

To understand whether the observed host–symbiont fidelity is the result of long-term cospeciation of *Pocillopora* with its *Cladocopium* symbionts, we performed a cophylogeny analysis by testing phylogenetic congruence using the procrustean approach^34^. The host–symbiont associations that contributed the most to the global phylogenetic congruence between *Pocillopora* and *Cladocopium* (i.e., lowest squared residuals) were the dependence of *P. grandis*, *P. meandrina*, and *P. verrucosa on C. latusorum* L4, L5, and *C. pacificum* respectively (Fig. 2b). Conversely, the weakest contributor to the global phylogenetic congruence was the dependence of *P*. cf. *effusa on C. pacificum* (L2) and SSH5_pver on *C. goreaui* and *C. pacificum* L3 in the central Pacific.

### Main drivers of holobiont gene expression variances across the Pacific Ocean

**Fig. 3:**
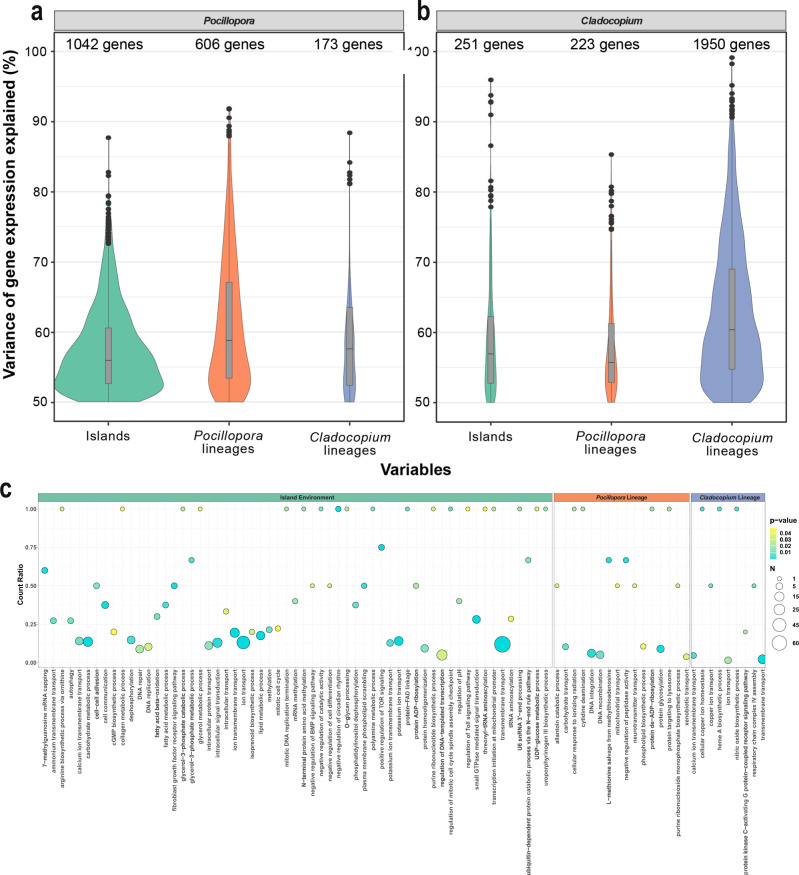
Contribution of environmental and genetic variables to *Pocillopora* and *Cladocopium* gene expression and functional enrichments of top variant genes. **a**, **b** Distribution of genes for which more than 50% of expression variation is explained by one of three predictor variables: sampled island, *Pocillopora* genetic lineage, and *Cladocopium* lineage. **a** Gene expression of *Pocillopora* across 101 samples (the hybrid colony was excluded). **b** Gene expression of *Cladocopium* across 70 samples. Samples of mixed lineage, from Rapa Nui island, and those containing *Durusdinium* were excluded. The number of genes in each distribution is indicated above each violin plot. Boxplots within violins display group medians (horizontal dark bar), interquartile range (bounds of box) and the minimum and maximum values (whiskers). Potential outliers are denoted as solid dots. **c** Gene Ontology enrichment analysis dot plots showing the top enriched biological process GO terms identified for each gene from comparison to a Wallenius noncentral hypergeometric sampling distribution allowing for *P* value calculation after accounting for selection bias and correction for multiple testing (FDR ≤ 0.05) using GOSeq (v1.40.0). *Pocillopora* genes with the highest explained variance under the island of sampling (left panel), host genetic lineage (middle panel), and photosymbiont lineage (right panel) variables are indicated. The *x* axis position and size of each dot reflects the proportion and absolute number of enriched genes (N) sharing that GO term, respectively, and the color of the dot indicates the enrichment significance. Source data are provided as a Source Data file.

The variation partitioning approach allowed us to identify genes whose expression levels were driven primarily by the sampling environment, the host genetic lineage, or the photosymbiont genotype (see “Methods”). For the host, among the 1821 genes with greater than 50% of their expression variation attributed to these three factors, 57% (1042 genes) were linked to the environment, 33% (606 genes) were associated with the host genetic lineage, and 9.5% (173 genes) were attributed to the photosymbiont genetic lineage (Fig. 3a). Functional annotations for the subset of these genes with corresponding annotations are presented in Supplementary Data 1. Similarly, we identified 2424 photosymbiont genes above the 50% explained variation threshold. In contrast to the host, 80% of these genes (1950 genes) are best explained by the genetic lineage of the photosymbiont, and only 10% (251 genes) and 9% (223 genes) of the genes are linked to the sampling island and the host lineage, respectively (Fig. 3b). Functional annotations for the subset of these genes with corresponding annotations are presented in Supplementary Data 2.

We investigated functional enrichments among these top variant genes and the results are presented in Supplementary Data 3–8. In the *Pocillopora* host, genes with expression dependent on the sampled island were enriched in biological process ontologies related to lipid and carbohydrate metabolism (*n* = 12 of 68 and 17 of 125 genes, respectively), ion transmembrane transport (16 of 82 genes) including transmembrane transport of ammonium (3 of 11 genes), and calcium (9 of 64 genes), as well as regulation of DNA repair (9 of 102 genes) and autophagy (3 of 11 genes; Fig. 3c and Supplementary Data 3). In *Cladocopium*, genes whose expression was dependent on the sampled island were associated with Pfam domains involved in heat shock response (HSP70, 2 genes), active transport of protons across membranes (E1–E2 ATPases, 2 genes), and oligosaccharide processing (Glucosidase II beta subunit-like, 1 gene; Supplementary Data 2).

Host genes whose expression was dependent on the *Pocillopora* genetic lineage were enriched in biological processes related to carbohydrate (3 of 29 genes) and xenobiotic transport (3 of 77 genes), DNA recombination (8 of 156 genes), and response to ionizing radiation (1 of 1 gene; Fig. 3d and Supplementary Data 5). *Cladocopium* genes whose expression was correlated with the photosymbiont lineage included Pfam enrichments related to ion transport (57 genes) and aquaporin-like or neutral solute transporters (Major intrinsic protein family, 9 genes; Supplementary Data 6).

*Pocillopora* genes whose expression was dependent on the photosymbiont lineage were enriched in biological process ontologies related to ion transmembrane transport (6 of 256 genes) particularly of calcium (3 of 64 genes), nitric oxide biosynthesis (1 of 1 gene), and assembly of the mitochondrial respiratory chain complex IV (1 of 2 genes; Fig. 3e and Supplementary Data 7). Among *Cladocopium* genes whose expression is dependent on the host, 14 are involved in carbohydrate metabolism (galactose oxidase, glucan synthase, glycosyl hydrolase) and gluconeogenesis (fructose-bisphosphate aldolase, enolase, phosphofructokinase-2). Thirteen calcium-transporter and calcium-dependent protein kinases that have been shown to play a role in the establishment of symbiosis^35^ are also dependent on the host lineage (Supplementary Data 2).

### Discriminant analysis of principal components (DAPC) of gene expression

To determine the main factors controlling gene expression variation in the host and the symbiont, we also performed a discriminant analysis of principal components (DAPC) of gene expression. For the host, the 11 PCs retained after a-score maximization in the DAPC explained 55.18% of total gene expression variance (Supplementary Fig. 4a). Discriminant axis eigenvalue scores were roughly equivalent between the first (DF1) and second (DF2) discriminant functions for the environmental grouping model (Supplementary Fig. 4b), but were notably higher for DF1 than for DF2 in the two other grouping models (Supplementary Fig. 4c, d). For the photosymbiont, the 11 retained PCs explained 73.86% of total gene expression variance (Supplementary Fig. 4e). Discriminant axis eigenvalue scores were highest for DF1 for all three grouping models (Supplementary Fig. 4f–h).

**Fig. 4:**
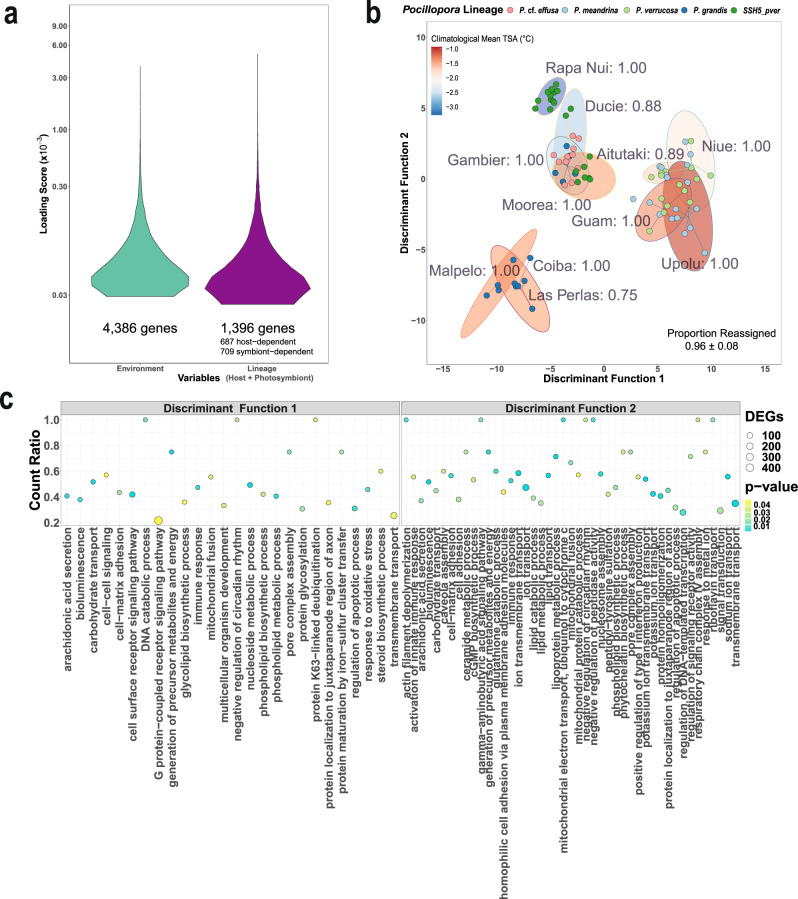
Discriminant analysis of principal components (DAPC) of *Pocillopora* host gene expression data grouped by the environment and biological process functional enrichments of top discriminant genes. **a** Loading scores of top discriminant genes revealed a roughly equivalent influence of the environment and of genetic lineage on gene expression in the *Pocillopora* host, but with a greater number of genes associated with the environment. **b** DAPC scatter plot showing expression profiles for the *Pocillopora* host when colonies were grouped by the environment. Points represent individual colony expression profiles and are colored by genetic lineage. Shaded ellipses denote 95%-confidence intervals around the group (island) mean and are colored by climatological mean thermal stress anomalies (TSA, 2002— sampling date). Group-specific proportions of correct reassignments are indicated within each cluster (labels) and the overall model proportion of correct reassignment (mean ± standard deviation) is presented in the bottom right. **c** Gene Ontology enrichment analysis dot plots showing the top enriched biological process GO terms identified for each gene from comparison to a Wallenius noncentral hypergeometric sampling distribution allowing for *P* value calculation after accounting for selection bias and correction for multiple testing (FDR ≤ 0.05) using GOSeq (v1.40.0). Data are shown for *Pocillopora* host genes contributing most strongly to the first (left panel) and second (right panel) discriminant function. The *x* axis position and size of each dot reflects the proportion and absolute number of enriched genes (DEGs) sharing that GO term, respectively, and the color of the dot indicates the enrichment significance. Source data are provided as a Source Data file.

For the *Pocillopora* host, when colony expression profiles were assigned to pre-defined groups according to their environment, host lineage, or photosymbiont lineage, we were able to correctly reassign 96%, 100%, and 99% of colonies to their respective groups (Supplementary Data 9). These groups were statistically distinct for all three models (Supplementary Data 10, all PERMANOVA *P* < 0.001). In *Cladocopium*, when colonies were assigned to pre-defined groups based on the environment, host lineage or photosymbiont lineage, we were able to correctly reassign 89%, 99%, and 99% of colonies to their respective groups (Supplementary Data 9). These groups were also distinct for all three photosymbiont models (Supplementary Data 10, all PERMANOVA *P* < 0.001). Therefore, based on proportions of colonies correctly re-assigned to pre-defined groups, our DAPC results revealed a marginally stronger influence of the genotype (or the genotype of the symbiotic partner) than of the environment for both the host and the symbiont. However, in the host we observed a higher number of genes associated with the environment (4386 genes) than associated with the host and symbiont lineages (1396 genes, Fig. 4a) in contrast to the symbiont where the number of genes were roughly equivalent (2495 for the environment and 2041 for the host and symbiont lineages, Supplementary Fig. 5a).

In the coral host under the environmental model, DAPC differentiated expression profiles by geography along DF1 and by environmental factors (especially historical thermal stress anomaly, TSA, severity) along DF2 (Fig. 4b). Colonies from the Eastern Tropical Pacific (Isla de Las Perlas, Coiba, and Malpelo) clustered around negative values of DF1 whereas colonies from the Western Pacific (Aitutaki, Niue, Upolu, and Guam) clustered at the positive end of this axis. Along DF2, colonies from islands with historically elevated SST and TSA values (Upolu, Guam, Isla de Las Perlas, Coiba, and Malpelo) clustered around negative DF2 values and colonies from the coldest islands (Rapa Nui and Ducie) clustered around positive values (Fig. 4b). In *Cladocopium* under the environmental model, DF1 primarily separated colonies from Rapa Nui from all others. The DF2 axis followed a similar pattern to that observed in the host, namely that colonies were distinguished from one another on this axis based on historical SST and TSA (Supplementary Fig. 5b).

We also investigated gene ontology enrichments among genes that contributed most strongly to the two discriminant functions in each environmental model (i.e., discriminant genes). The results of these analyses are presented in Supplementary Data 3–8. Under the environmental model, host genes which discriminated between islands along DF1, the geographical axis, were enriched in biological processes related to carbohydrate binding and transport (27 and 15 genes, respectively) and glycolipid biosynthesis (13 genes, Fig. 4c). Host genes which discriminated along DF2, the TSA axis, were enriched in processes related to protein ubiquitination and repair (32 and 2 genes, respectively) and mitochondrial respiratory chain complex assembly (four genes, Fig. 4c). Functional enrichments shared across both axes included processes related to ion transmembrane transport (28/45 genes for DF1/DF2, respectively), generation of precursor metabolites and energy (2/1/4 genes for DF1/DF2/shared, respectively), immune response (3/4/7 genes), response to oxidative stress (2/3/8 genes), and cytolysis in other organism involved in symbiotic interaction (3 shared genes). In *Cladocopium*, genes whose expression strongly distinguished photosymbionts from Rapa Nui from all others (DF1 contributing genes) were enriched in protein family (Pfam) domains related to chlorophyll A-B binding (35 genes) and ion transport (91 genes; Supplementary Data 4). *Cladocopium* genes which contributed to DF2 were not significantly enriched in any Pfam domains (Supplementary Data 4). DAPC models based on the host and photosymbiont lineages are presented in the Supplementary Results (section 1) and in Supplementary Figs. 5c, d, 6, and 7.

### Top environmental variables contributing to gene expression divergence (CCA)

**Fig. 5:**
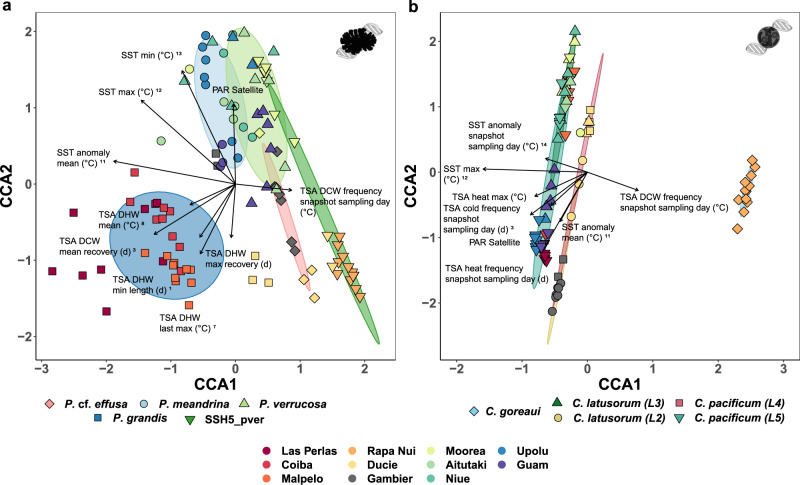
Constrained correspondence analysis (CCA) of *Pocillopora* host and *Cladocopium* symbiont gene expression profiles from colonies collected across the Pacific Ocean. Scatter plots showing (**a**) *Pocillopora* host (coral colony icon in upper right) and **b** *Cladocopium* photosymbiont (Symbiodiniaceae icon in upper right) expression profiles for individual colonies with data colored by island and clustered by genetic lineage (i.e., *Pocillopora* SVD clade or *Cladocopium* genetic lineage; shaded ellipses). Length (strength) and direction (towards increasing values) of vector arrows denote the relative influence of the top explanatory environmental variables for each dataset. For variables that were strongly correlated (*r*^2^ ≥ 0.7), a single representative is listed alongside a numeric superscript denoting the corresponding correlation cluster in Supplementary Fig. 8. Descriptions of top environmental variable abbreviations are given in Supplementary Data 11. Source data are provided as a Source Data file.

Using automatic stepwise selection of the top explanatory environmental variables for constrained correspondence analysis (CCA), we identified 10 environmental variables (or variable clusters) that best explained the dispersion among host gene expression profiles (Fig. 5a, Supplementary Data 11, and Supplementary Fig. 8). Similarly, for the photosymbiont, we identified 8 environmental variables (or clusters) that best explained the gene expression dispersion (Fig. 5b, Supplementary Data 11, and Supplementary Fig. 8). For the coral host, climatological degree heating week (DHW) associated data were among the strongest contributors to gene expression variation between colonies whereas in the *Cladocopium* photosymbiont, mean climatological SST associated data were the strongest contributors (Supplementary Data 11). The genetic lineage also contributed strongly to expression profile differences in both the host and photosymbiont. In the *Pocillopora* host, separation of gene expression profiles along both constrained correspondence axes (CCA1 and CCA2) was primarily driven by differences in degree heating week severity between sampling locations. Colonies from islands with low DHW values (e.g., Rapa Nui) clustered around positive CCA1 values, whereas colonies from islands with historically elevated SSTs (e.g., Upolu) clustered around negative CCA1 values (Fig. 5a). In contrast, CCA2 was primarily tied to SST variability (Fig. 5a). Negative CCA2 values were associated with islands with the highest maximum surface temperature anomalies (e.g., Las Perlas and Coiba) or those with extreme DHW events. Conversely, positive CCA2 values were associated with islands with long recovery periods between extreme heating events (i.e., large degree heating week recovery period, e.g., Rapa Nui) and/or long degree cooling week durations (e.g., Moorea).

In the *Cladocopium* photosymbiont, historical SST was strongly negatively correlated with CCA1 (Fig. 5b). Colonies from cooler islands (e.g., Rapa Nui) clustered around positive CCA1 values whereas those from warmer locations tended to cluster around negative CCA2 values (e.g., Upolu). As in the coral host, CCA2 was primarily associated with DHW intensity. Colonies from islands with elevated DHW exposure tended to cluster around negative CCA2 values. Conversely, colonies from islands with prolonged exposure to cooling events clustered together around positive CCA2 values (Fig. 5b). These results suggest that whereas gene expression profiles in both the coral host and photosymbiont are primarily structured by climatological SST, it is the frequency of exposure to extreme heating/cooling events that drives substructuring of gene expression in the host/photosymbiont, respectively.

### Genes differentially expressed between *Cladocopium*- and *Durusidinium*-containing colonies

According to unconstrained analysis, global gene expression profiles of colonies from the Eastern Tropical Pacific (Isla de Las Perlas, Coiba, and Malpelo) differed between islands (PERMANOVA, *P* ≤ 0.05), but not between *Cladocopium*- and *Durusdinium*-containing colonies (Supplementary Fig. 9, PERMANOVA, *P* > 0.05). In addition, intra-island comparison of expression profiles between colonies in the Eastern Tropical Pacific revealed only a small number of genes that were differentially expressed between *Cladocopium*- and *Durusdinium*-containing hosts (Supplementary Data 12). We observed 294 ± 363, 129 ± 53, and 110 ± 15 genes upregulated in *Durusdinium*-containing colonies (FDR-adjusted *P* ≤ 0.05 and a LFC ≥ 2) on the three islands, respectively (Supplementary Fig. 10a). We also observed 171 ± 164, 211 ± 102, and 200 ± 59 genes downregulated in *Durusdinium*-containing colonies (FDR-adjusted *P* ≤ 0.05 and a LFC ≤ −2) on the three islands, respectively (Supplementary Fig. 10b). On average, we found 129 genes differentially expressed between *Cladocopium*- and *Durusdinium*-containing *Pocillopora* colonies in the Eastern Tropical Pacific (Supplementary Data 10) with very few DEGs shared between pairs of islands. The number of pairwise shared DEGs ranged from three (upregulated in both Las Perlas and Coiba) to nine (downregulated in both Las Perlas and Coiba) and no gene was commonly differentially expressed across all three islands (Supplementary Fig. 10a, b and Supplementary Data 12).

We also examined functional enrichments among genes differentially expressed between *Cladocopium*- and *Durusdinium*-containing colonies and these results are listed in Supplementary Data 4. In general, genes upregulated in *Durusdinium*-containing colonies on Las Perlas, were enriched in GO biological processes related to bioluminescence, xenobiotic transport, gluconeogenesis, mitochondrial protein catabolic process, base-excision repair, and transmembrane transport (Supplementary Data 12). Genes downregulated in *Durusdinium*-containing colonies on Las Perlas, were enriched in GO biological processes related to calcium ion transmembrane transport, and phospholipid biosynthetic processes. Genes upregulated in *Durusdinium*-containing colonies on Coiba, were enriched in GO biological processes related to protein phosphorylation and proteolysis whereas downregulated genes were enriched in processes related to chaperone-mediated protein folding, chaperone-mediated protein transport, synaptic vesicle transport, nuclear envelope organization, protein phosphorylation, cell adhesion, neuron projection development, and intermediate filament cytoskeleton organization (Supplementary Data 12). Finally, genes upregulated in *Durusdinium*-containing colonies on Malpelo, were enriched in GO biological processes related to bioluminescence, regulation of signaling receptor activity, vacuolar transport, and xenobiotic transport (Supplementary Data 12). Genes downregulated in *Durusdinium*-containing colonies on Malpelo, were enriched in GO biological processes related to bioluminescence, regulation of apoptotic process, protein phosphorylation, S-adenosylmethionine biosynthesis, regulation of pH, notch signaling pathway, MyD88-dependent toll-like receptor signaling pathway, proteolysis, signal transduction, protein ubiquitination, and netrin-activated signaling pathways.

## Discussion

### *Pocillopora* corals exhibit high symbiotic fidelity with distinct *Cladocopium* lineages

Strong fidelity between genetically distinct coral hosts and specific Symbiodiniaceae species has been described for a wide variety of reef-building species^36–38^ including pocilloporids^39,40^, although exceptions do exist^41^. Across the Pacific ocean, we found evidence for near perfect fidelity for three *Pocillopora* species (*P*. cf. *effusa*, *P. meandrina*, and *P. verrucosa*), and limited flexibility for two species (*P. grandis* and SSH5_pver). For *P. grandis* and SSH5_pver, their two *Cladocopium* symbionts match to two distinct subclades that are geographically separated (discussed below). *P. grandis*/*P. meandrina* associated with *C. latusorum* and *P. verrucosa* with *C. pacificum* in concordance with previous observations^29,33^. The association of SSH5_pver with *C. goreaui* or *C. pacificum* and *P*. cf. *effusa* with *C. pacificum* has not been previously reported.

Our findings, therefore, support the general consensus that fidelity to a single putative symbiont species is the dominant pattern in most scleractinian corals^42^ including those inhabiting some of the hottest reefs in the world^13,43,44^. Previous studies have suggested that this tendency for high symbiotic fidelity in corals, and within pocilloporids specifically, reflects an underlying cospeciation between the *Pocillopora* host and its photosymbiont^29,33^, which supports the persistence of distinct holobiotypes which maximize holobiont fitness in a given environment.

Despite this overarching tendency towards symbiotic fidelity in corals, symbiotic associations are also sometimes flexible indicating a potential role for acclimatory phenotypic plasticity to modulate holobiotypes under certain conditions, particularly acute heat stress^12,22,41,45^. We detected two broad instances of symbiont flexibility among the sampled *Pocillopora* species including a differential association with *D. glynnii* among *P*. grandis hosts in the Eastern Tropical Pacific (Isla de Las Perlas, Coiba, and Malpelo islands) and within a single colony of *P. verrucosa* on Guam. *Pocillopora* - *Durusdinium* associations have been reported on previously. For example, in the far Eastern Tropical Pacific, *P. meandrina* (mtORF type 1) and *P. verrucosa* (mtORF type 3) hosts are known to flexibly alter their symbiotic associations between certain lineages of *Cladocopium* and *D. glynnii* depending on local thermal conditions^39,41^. Similarly, on Guam, *Pocillopora* colonies hosting *D. glynnii* were shown to have better maintained photosynthetic productivity under acute heat stress than those hosting *Cladocopium*^46^. Because these associations appear to be stable and persistent in these locations across time, it can be argued that this photosymbiotic association is stable between pocilloporids and *D. glynnii*, particularly in the far Eastern Tropical Pacific where the association is common, may indicate a stable strategy for survival on these warm reefs as discussed below.

The association of one *Pocillopora* species with two different *Cladocopium* lineages was always observed in geographically distant islands supporting the hypothesis of speciation driven by niche diversification within *Cladocopium* genera^33^. In addition, this flexibility of symbionts matches perfectly with two different subclades of each *Pocillopora* species. Therefore this apparent flexibility is probably stable and not due to recent changes in the environment.

In general, the *Pocillopora* phylogeny is similar to the *Cladocopium* phylogeny suggesting that the symbiont speciation is driven by the host in agreement with the cospeciation hypothesis^29^. However, we observed two major breaks in this cospeciation not previously reported. In the first instance, the association of SSH5_pver with two genetically distant *Cladocopium* could be due to the relative isolation of SSH5_pver colonies from Rapa Nui and consequent adaptation to the unique local environmental conditions in this location (e.g., cool SSTs). The presence of *C. pacificum* in *P*. cf. *effusa* in the central Pacific is also incoherent with the cospeciation pattern, but the reasons for this difference in symbiotic association remain to be studied.

### Coral host transcriptomic plasticity maintains regulatory homeostasis across environments

Altered gene expression can act to maintain symbiotic homeostasis under changing environmental conditions (i.e., transcriptomic plasticity). However, assessing coral holobiont transcriptomic plasticity is complicated by the fact that many different drivers (e.g., genetic diversity, environmental stressors, and biotic associations) may interact to determine expression profiles in natura. For the *Pocillopora* host, we identified a greater number of genes whose expression profiles were correlated with variation in the environment than with either the host- or symbiont-genetic lineages. However, both analyses also indicated that the most influential genes (i.e., those with the highest explained variance and/or loading scores) had expression profiles that were linked to the host genetic lineage. This mixed result, suggests that the steady-state expression profiles we measured in *Pocillopora* hosts are jointly responsive to the host genetic lineage and to the local abiotic environment. Host gene expression therefore reflects the classical conception of the phenotype as an integrated expression of the genotype and the environment. The high proportion of colonies that were correctly re-assigned to their genetic lineage based on their expression profiles (100%, Supplementary Fig. 6b) in the lineage-informed DAPC model further implies that, in the *Pocillopora* coral, the host genotype acts to establish a phenotypic baseline around which expression profiles vary in response to the local abiotic environment.

Although they represented only a small fraction of the top variant genes, *Pocillopora* genes with expression dependent on the symbiont lineage were involved in several important biological processes including those potentially related to regulation of calcification, symbiont photosynthesis, and to the scavenging of reactive oxygen species. Among them, ATPase-coupled transmembrane transporters are known to play a role in host calcification^47–49^ and as carbon-concentrating mechanisms in marine photosymbioses thereby regulating *Symbiodiniaceae* photosynthesis^47,50–53^. These responses likely serve to maintain symbiosome homeostasis and regulate photosymbiont productivity across environments.

### Photosymbiont transcriptomic profiles are primarily driven by algal genotype

In contrast to the host, gene expression profiles within *Cladocopium* were most strongly correlated to their respective genetic lineages and comparatively weakly influenced by the environment. We suggest two, potentially complementary, explanations for the difference in environmental sensitivity between the host and the symbiont: (i) it reflects a primarily host-driven homeostatic regulation of the photosymbiont micro-environment such that environmental influences on symbiont expression are minimized, and/or (ii) that symbiont transcriptomic profiles reflect inherent differences in physiological regulation between microalgal species which outweighed environmental influences over the range of thermal habitats we investigated in this study.

Previous research has suggested that Symbiodiniaceae themselves often exhibit relatively invariant expression profiles in response to environmental change^54,55^. For example, following a simulated heatwave event, *Acropora aspera* hosts exhibited a significantly more robust transcriptional response than their associated photosymbionts with strong upregulation of several host genes including heat shock proteins^54^. In addition, bleaching susceptibility of corals following an acute heatwave was found to be tied to a reduced capacity of the host to maintain intracellular acid–base homeostasis^56^. Finally, in photosymbiotic *Aiptasia*, disruption of host homeostasis occurred prior to symbiont photoinhibition and bleaching suggesting that it is the cnidarian host that first responds to environmental perturbation^57^. Further research is necessary to determine whether the relatively stable gene expression profiles we observed within *Cladocopium* lineages across environments are a result of strong regulatory control by the *Pocillopora* hosts and the degree to which these profiles may exhibit plasticity under acute thermal stress.

### Convergent host plasticity and its limitations under elevated SST

Despite our finding that genotypic effects may, in general, outweigh plastic responses among *Pocillopora* corals, we did observe a significant response to the environment among host lineages. Constrained correspondence analysis of expression variation between islands indicated that the strongest environmental factor contributing to colony gene expression was historical SST, specifically degree heating week exposure. This is coherent with the primacy of temperature as an abiotic driver in ectothermic organisms generally^58^ and given its specific, disruptive, effects on regulatory homeostasis within scleractinian corals^3,19^. Colonies from different genetic lineages and from geographically distant locations nevertheless displayed similar expression profiles linked to thermal stress anomaly (Fig. 4b, DF2). This suggests a convergent transcriptomic response to thermal challenge across *Pocillopora* lineages. Interestingly, these same colonies also displayed high fidelity for three specific photosymbiont lineages—*C. latusorum* L3 and *C. pacificum* L5 as well as *Durusdinium glynnii* - which may provide further evidence for thermal specialization and/or coevolution in these species.

In terms of coral host transcriptomic plasticity, among genes whose variation in expression was principally correlated with the local environment, we observed an upregulation of genes traditionally associated with the environmental stress response (ESR) in colonies present on historically warm reefs. Host lineages from warm islands showed elevated expression of Heat Shock Protein Family A (HSP70) Member 12A-like. This class of chaperones is known to be involved in response to thermal stress in scleractinian corals^59^. On these islands, SSTs were not abnormally elevated at the time of sampling and colonies had not experienced an extreme heating event in the previous 12 weeks before sampling, suggesting that this elevated expression of ESR genes among these colonies are indicative of gene frontloading, a potential signal of local thermal adaptation. In addition, we observed enrichment among environmentally responsive genes in xenobiotic transporters including both glutathione and lipid peroxidase activities. These functions may represent upregulation of membrane-bound GSTs (MAPEG), an evolutionarily distinct class of enzymes that detoxify xenobiotic compounds and ameliorate oxidative stress^60^.

### Differences in symbiotic association under elevated sea surface temperatures

The coral holobiont is composed of a diverse community of organisms which potentially display differing sensitivities to warming. Shifts in the composition of the holobiont can therefore be as important as shifts in gene expression for permitting acclimatization to thermal challenges^61,62^. Such shifts can occur over long time periods as a result of competitive exclusion between photosymbiont lineages within a colony^44^, or over relatively short time scales in response to acute stress^45,63^. However these short-term changes are usually reversibly plastic, typically reverting when the stressful conditions cease^40,63,64^. Both mechanisms can facilitate acclimatization to the local thermal environment, as has recently been shown in photosymbiotic giant clams^65^. Although the *Pocillopora* lineages we sampled typically exhibited high symbiotic fidelity with specific *Cladocopium* lineages, this was not always the case.

In total, we observed 23 instances in which *Pocillopora* lineages hosted different symbiont communities under different environmental contexts. In these instances, host colonies either exhibited a difference in association between specific *Cladocopium* photosymbionts in different environments or were dominated by *Durusdinium glynnii* photosymbionts. Interestingly, in all these cases, when a given coral host lineage was present on multiple islands that spanned a thermal gradient, we saw a *preferential association* with one of three photosymbionts (*Cladocopium pacificum* L3 and *C. latusorum* L5, or *Durusdinium glynnii*) on the warmer island(s). In fact, with the exception of a single *P*. cf. *effusa* (SVD1) colony on Moorea which hosted *C. pacificum* L2 symbionts, we always observed a preferential association with *C. pacificum* L3 and C. *latusorum* L5 in *Pocillopora* colonies inhabiting islands with mean historical SSTs >26.8 °C. This observation suggests that whereas pocilloporid corals typically exhibit symbiotic fidelity, they are capable of a limited form of symbiotic flexibility involving preferential association with specific lineages of *Cladocopium* (and *Durusdinium*) depending on the local environment^66,67^. This difference in association may serve to maximize holobiont fitness under elevated SST and suggests a potential strategy for community-driven thermal acclimatization.

Finally, we also observed 14 *Pocillopora* colonies which were either simultaneously hosting two photosymbiont genera or which contained *Durusdinium glynnii* symbionts as the dominant community member (Fig. 1b). These colonies were largely collected from islands in the Eastern Tropical Pacific (Las Perlas, Coiba, and Malpelo islands) which have historically experienced prolonged (mean sea surface temperatures >26.8 °C) and/or severe warming (highest thermal stress anomalies, TSAs, among the sampled islands). A similar pattern has been reported on extensively, both in the Eastern Tropical Pacific^39,41^ and more broadly on warm reefs globally^22,68–70^. For example, in *Montipora capitata* corals within the Hawaiian archipelago which displayed higher proportions of *Durusdinium* symbionts on reefs with higher frequencies of thermal stress anomalies (TSAs)^71^. Although the prevalence of *Durusdinium* in our data was not correlated with TSA frequency, it was strongly correlated with TSA magnitude suggesting the potential for photosymbiont shuffling/switching in these colonies in response to a recent or historical thermal challenge^72^. Alternatively, because *Pocillopora meandrina* symbionts are generally acquired via vertical transmission, with oocytes being seeded with the maternal symbiont community^73^, it is also possible that these *Cladocopium*- and *Durusdinium*-containing colonies represent true, evolutionarily stable, holobiotypes which persist across generations on reefs which experience frequent and/or severe thermal stress. However, whereas it has been suggested that some corals can increase their heat tolerance limits by +1.0–1.5 °C following a change in their symbiont community composition (often resulting in domination by *Durusdinium trenchii*, formerly D1a), repeated or prolonged exposure to high temperature often still results in colony mortality^19,23,45,74^. Thus, the degree to which the specific association between pocilloporid corals and *Durusdinium* under elevated SSTs confers an adaptive advantage under prolonged future warming requires further investigation.

### Limited transcriptomic response of the host under different symbiotic associations

Despite the increased prevalence of *Durusdinium* symbionts in *Pocillopora* colonies on warm reefs, especially in the far Eastern Tropical Pacific, this difference in association had very little effect on the host gene expression we measured in natura. Our data indicate that photosymbiont community composition (i.e., dominance of *Cladocopium latusorum* L5 or *Durusdinium glynnii*) had only a weak, and inconsistent, impact on host expression and that host expression was driven more by the environment than by photosymbiont community composition. This stands in contrast to previous studies which have reported a significant role of the photosymbiont in modulating coral host gene expression^75^. For example, a recent study in *Montastraea cavernosa* reported that colonies containing *Durusdinium glynnii* symbionts displayed elevated expression of ESR genes even under ambient conditions relative to colonies containing *Cladocopium* symbionts^22^. These results suggest that photosymbiont genotype significantly alters host expression even prior to environmental perturbation.

In this study, only 4% of gene expression variation between Eastern Tropical Pacific coral colonies was attributed to differences in the dominant photosymbiont whereas in *M. cavernosa* colonies up to 14% of expression variation between individuals could be attributed to the switch between dominant symbiont lineages^22^. The environment played a much more significant role in determining *Pocillopora* gene expression in our dataset with 24% of expression variation being explained by the sampling island. Additionally, we observed very little to no overlap in differentially expressed genes between *Cladocopium*- and *Durusdinium*-containing *Pocillopora* colonies across the three islands (Supplementary Fig. 10). This implies that there was no common *Durusdinium*-dominance effect on host expression across environments. Finally, we observed high gene expression variation among *Durusdinium*-containing *Pocillopora* colonies from within the same reef (Supplementary Figs. 9 and 10) suggesting that, even at small spatial scales, other factors were more important than photosymbiont lineage in determining host gene expression.

Despite the weak influence of the dominant symbiont lineage on overall *Pocillopora* host expression in the Eastern Tropical Pacific, we did observe a small set of genes that were differentially expressed between *Cladocopium*- and *Durusdinium*-containing hosts. These included genes associated with chaperone-mediated protein folding, protein ubiquitination, and regulation of apoptosis which were among the most significantly enriched biological process terms associated with genes downregulated in *Durusdinium*-containing colonies (Supplementary Data 12). These results provide indirect support for potential thermal acclimatization in that coral colonies containing putatively thermally tolerant photosymbionts (e.g., *Durusdinium*) showed reduced expression of ESR.

Thus, in our dataset, we observed three photosymbiont lineages (*C. pacificum* L3, *C. latusorum* L5, and *D. glynnii*) which appear to have been in preferential association with *Pocillopora* hosts inhabiting islands with historically elevated SSTs and/or TSAs. Although non-selective mechanisms could account for this pattern (e.g., dispersal limitations resulting in the dominance of particular photosymbionts on nearby reefs, particularly if those reefs are geographically isolated), these mechanisms seem unlikely in this study. The fact that we observed the same photosymbiont communities in colonies inhabiting reefs ca. 14,000 km apart across the Pacific Ocean (e.g., Coiba and Guam) and under very similar environmental conditions (i.e., mean historical SSTs >26.8 °C) suggests that the high holobiont fidelity and specificity we observed among multiple lineages of *Pocillopora* corals is reflective of a process of cospeciation between *Pocillopora* hosts and their photosymbionts. Given the similar strong patterns of cophylogeny between *Pocillopora* and *Cladocopium* we observed and the limited environmental plasticity of photosymbiont expression across the Pacific Ocean, we interpret this strategy as one relying on host-mediated transcriptomic plasticity to buffer the holobiont from environmental change alongside preferential association with specific, thermotolerant, *Cladocopium* lineages under elevated SSTs. However, at extreme SSTs/TSAs even these heat-tolerant *Cladocopium* lineages are ultimately replaced by *Durusdinium D1* photosymbionts suggesting there may be upper limits to the effectiveness of this strategy under warming. Whereas high levels of gene flow between populations across large spatial scales in both *Pocillopora* hosts and their photosymbionts^27,29^ has been suggested as an beneficial trait for permitting adaptation to previous climate shifts^29^, the degree to which this strategy of high fidelity and specificity between *Pocillopora* hosts and their photosymbionts may act to aid or hinder their adaptation to predicted future warming warrants further research.

Overall, we observed high symbiotic fidelity among *Pocillopora* lineages across environments with evidence of potential selection for heat-resistance photosymbiont lineages on islands with historically elevated sea surface temperatures. We reveal that host gene expression profiles are strongly segregated by host genetic lineage and environment, and were significantly correlated with several historical sea surface temperature (SST) traits. In contrast, *Cladocopium* expression profiles were primarily driven by algal genotype and displayed low phenotypic plasticity across environmental gradients. Overall, our data suggest the existence of a two-tiered strategy underpinning thermal acclimatization in *Pocillopora* holobionts with strong selection for specific photosymbiont lineages (i.e., host–photosymbiont fidelity) coupled with high host transcriptomic plasticity acting as an environmental buffer.

We observed photosymbiont flexibility only in association with elevated SSTs in the far Western and far Eastern Pacific. Although the thermotolerant photosymbiont *Durusdinium glynnii* was largely absent from *Pocillopora* colonies in the Central Pacific and rare in the West Pacific (only two colonies) in our dataset, previous studies have shown that *D. glynnii* frequently occurs in *Pocillopora* corals in many locations in the Pacific, particularly those with elevated SSTs (more than 31.5 °C) or which repeatedly experience extreme thermal events^12,46,69,70,76^. Taken together, our results and previous studies show that the presence of *D. glynnii* photosymbionts is a common strategy for *Pocillopora* corals to survive heat stress.

Our study has important implications for continued coral reef conservation and the prediction of coral responses to future ocean warming. Our examination of the mechanisms underpinning acclimatization among lineages across an environmental gradient and our identification of potential candidate loci under thermal expression selection provide useful baseline metrics for informing future manipulative experiments focused on physiological mechanisms underlying these observations. Investigating the extent to which different thermal acclimatization strategies, including both transcriptional and compositional changes, are employed among lineages will allow us to better forecast the survival of coral species and reefs under future ocean warming.

## Methods

### Site selection, coral colony sampling, and environmental metadata collection

A total of 102 *Pocillopora* spp. colonies from 32 reef sites across 11 islands (Islas de las Perlas, Coiba, Malpelo, Rapa Nui, Ducie, Gambier, Moorea, Aitutaki, Niue, Upolu, and Guam) were sampled as part of the *Tara* Pacific expedition^26^. At each island, fragments of three coral colonies were collected from each of three reef sites yielding a total of ca. 9 colonies sampled per island (Supplementary Data 13). To reduce variation in host/symbiont expression associated with diurnal cycles, all sites were sampled between ca. 8:30–11:30 in the morning (local time). Colonies were sampled at a mean depth of 9.20 ± 3.80 m (SD; Supplementary Data 13). Reef site and coral colony sampling protocols are presented in detail in ref. ^77^. Fragments of sampled coral colonies were removed from the reef and brought back on board the *Tara* vessel for processing of DNA/RNA. Several environmental parameters such as SST, chlorophyll concentration, pH, and nutrient concentrations were measured in natura at the time of collection^78^. Historical sea surface temperature (SST) data were extracted from day and night satellite measurements of SST at 1 km spatial resolution from 2002 to the day of sampling as described in ref. ^77^ (Supplementary Data 13). Briefly, SST climatologies, anomalies, and several other variables including degree heating week data, frequency, and recovery time were calculated from daily averages of three satellites sensors (MODIS-Aqua, MODIS-Terra, VIIRS-SNPP) over the period studied^79^. Each variable average, standard deviation, and maximum were then calculated to provide one value per sampling site.

### DNA/RNA isolation, library preparation, and sequencing

Coral fragments were processed to extract and isolate DNA/RNA as described in ref*.* ^80^. Briefly, coral fragments were submerged in 15 mL of lysing agent in the presence of DNA/RNA shield and coral tissue was removed from the skeletal fragments and homogenized using a high-speed bead homogenizer. The homogenized tissue suspension was split into ten aliquots of 500 µL and stored in 1.5-mL Eppendorf tubes at −20 °C until extraction of nucleic acids. DNA and RNA were extracted simultaneously from these aliquots using Quick-DNA/RNA Kits (Zymo Research, CA, USA). DNA and RNA were quantified on a Qubit 2.0 fluorometer with Qubit dsDNA BR (Broad range) and HS (High sensitivity) Assays and Qubit RNA HS Assay (ThermoFisher Scientific, Waltham, MA, USA), respectively. The quality of total RNA was checked by capillary electrophoresis on an Agilent Bioanalyzer using the RNA 6000 Pico LabChip kit (Agilent Technologies, Santa Clara, CA) and the RIN was calculated. Eluted DNA and RNA were stored at −20 °C and −80 °C, respectively, prior to library construction.

Construction of DNA libraries began by shearing to a mean size of 300 bp using a Covaris E210 instrument (Covaris, Inc., USA). Size profiles of sheared materials were visualized on an Agilent Bioanalyzer DNA High Sensitivity chip, and sheared DNA was then end-repaired, 3’-adenylated, and ligated to Illumina compatible adapters using the NEBNext DNA Modules Products (New England Biolabs, MA, USA) and NextFlex DNA barcodes (Bioo Scientific Corporation). After two consecutive cleanups using 1× AMPure XP bead, DNA was amplified using Kapa Hifi HotStart NGS library Amplification kits (Kapa Biosystems, Wilmington, MA), followed by 0.6× AMPure XP bead purification. DNA libraries were stored at −20 °C until sequencing.

RNA libraries were prepared following the TruSeq Stranded mRNA sample preparation protocol. Extracted RNA (1 µg) was subjected to poly-A+ selection using oligo(dT) beads, and the resulting mRNA was chemically fragmented under elevated temperature before conversion into single-stranded cDNA using random hexamer priming. Then, the second strand was generated to create double-stranded cDNA. Double-stranded cDNA was purified by 1.8× AMPure XP bead cleanups, 3’-adenylated, and ligated to TruSeq RNA barcoded adapters with 6 bases (Illumina, San Diego, CA, USA) or NEXTflex DNA barcoded adapters with 12 bases (Bioo Scientific, Austin, TX, USA). After another 1× AMPure XP bead cleanup, the ligated product was amplified by 15 cycles of PCR and purified by a final 0.8× AMPure XP bead cleanup. RNA libraries were stored at −80 °C until sequencing.

Size profiles of DNA and RNA libraries were generated using an Agilent 2100 Bioanalyzer (Agilent Technologies, USA) and libraries were quantified by qPCR with the KAPA Library Quantification Kit for Illumina Libraries (Kapa Biosystems) on an MXPro instrument (Agilent Technologies). DNA (i.e., metagenomic) and RNA (i.e., metatranscriptomic) libraries were sequenced using 151 bp paired-end read chemistry on a NovaSeq or HiSeq4000 Illumina sequencer (Illumina, San Diego, CA, USA). Basecalling and calculation of Phred quality scores (Q scores) were performed during sequencing by the Illumina Real Time Analysis (RTA) software. Illumina bcl2fastq Conversion software converted raw BCL files generated by RTA to fastq data. For both metagenomic and metatranscriptomic reads, we removed short (<30 bp length) and low-quality nucleotides (Q score <20), adapter/primer sequences with an in-house script based on Fastx-Toolkit software (https://github.com/institut-de-genomique/fastxtend), as well as read pairs that mapped to the Enterobacteria phage PhiX174 genome (GenBank: NC_001422.1). For metatranscriptomic reads, read pairs that mapped to ribosomal sequences were removed using SortMeRNA v2.1^81^. After this filtering, we obtained between 39 M and 104 M metatranscriptomic reads (Supplementary Data 14) and between 81 M and 221 M metagenomic reads (Supplementary Data 15).

To identify Symbiodiniaceae lineages, we amplify the ITS2 region of the nuclear ribosomal DNA locus from Coral DNA extractions using SYM-VAR-5.8S2 (5’-GAATTGCAGAACTCCGTGAACC-3’) and SYM-VAR-REV primers (5’-CGGGTTCWCTTGTYTGACTTCATGC-3’). Complete protocols for DNA amplification, library preparation and sequencing of ITS2 amplicons are available in ref. ^80^. ITS2 reads were processed following the SymPortal pipeline^82^ and raw results are available^83^.

### Host lineage assignation

We performed host lineage assignment as described in ref. ^27^. Briefly, we identified a set of genome-wide single nucleotide polymorphisms (SNPs) from metagenomic reads mapped to the *Pocillopora meandrina* genomic reference^84^ using the Genome Analysis Toolkit tool (GATK, v3.7.0)^85^. We followed a modified version of the best practices guide for variant discovery with GATK which included indexing of the genomic reference (picardtools v2.6.0, CreateSequenceDictionary), followed by identification of realignment targets (GATK RealignerTargetCreator) and realignment around detected indels (GATK, IndelRealigner). Variants were called for each colony individually (GATK, HaplotypeCaller) and resulting variant call files (VCFs) were merged into island-specific, multi-sample, cohort files (GATK, CombineGVCFs) before performing joint genotyping across all 11 islands (GATK, GenotypeGVCFs) with polyploidy defined at 1^86^. Joint analysis of multiple samples (i.e., joint genotyping) is recommended for discovery of germline SNPs and indels as it provides information regarding population-wide variance across a cohort of multiple samples. This, in turn, allows for a higher degree of sensitivity to detect low-frequency variants, a clearer distinction between homozygous and missing sites, a greater ability to avoid false positives, and results in more accurate sample genotyping as described in the GATK technical documentation^87^. The resulting SNPs were filtered using VCFtools (v0.1.12)^88^ to include only biallelic sites with minor allele frequencies ≥0.05, quality scores ≥30, coverage (minimum read depth) ≥16, linkage disequilibrium (*r*^2^) ≥ 0.2, and no missing data across colonies. These curated SNPs were then used to identify divergent host lineages using a coalescent analysis performed on the individual colonies as described in ref. ^27^ using RaxML.

### *Cladocopium* lineage assignation

To determine which *Symbiodiniaceae* taxa were present in each coral colony, we used ITS2 amplicons determined for each sample. ITS2 profiles were obtained using SymPortal^82^, which uses the intragenomic diversity of Symbiodiniaceae ITS2 to define so-called ITS2 type profiles based on consistent co-occurrence of intragenomic ITS2 variants across all samples.

For *Pocillopora* colonies hosting *Cladocopium* (84 colonies), we further investigated their population structure using single nucleotide polymorphism (SNP) distributions across their coding sequences. We excluded from these analysis colonies containing a large proportion (>25%) of a second ITS2 profile and potentially affecting the SNP calling. SNPs from the resulting 82 colonies were identified from metatranscriptomic reads previously aligned on the predicted coding sequences of the *Cladocopium goreaui* genome (see next paragraph) following the same protocol as described for the host above. SNPs were filtered using VCFtools (v0.1.12)^88^ to include only biallelic SNPs with a quality score ≥30 and a coverage ≥4. The frequencies of these SNPs were then clustered using the Hclust function of the stats package (v4.2.2) in R with the completeLinkage method. The optimal number of clusters was determined with the gap statistics method^89^ (fviz_nbclust function in the R package factoextra v1.0.7). We then compared the Bray-Curtis-derived pairwise distances (based on ITS2 type profiles) of the *Cladocopium*-containing *Pocillopora* colonies to the SNP-based clustering of *Cladocopium* populations. In general, we observed a high degree of overlap between the two identification methods across *Cladocopium* lineages. The ITS2 profile distances indicate that *Cladocopium* lineages which were undefined in the SNP clustering method belong to L5 (Supplementary Fig. 2b).

Finally, we extracted reads matching the non-coding region of the psbA gene (psbA^ncr^) from metagenomes to assign *Cladocopium* lineages to *Cladocopium* species previously identified^29,33^. For this analysis, metagenomic reads of the 82 *Pocillopora* corals containing *Cladocopium* symbionts were aligned simultaneously on three psbA^ncr^ sequences (*C. latusorum* MW819767.1, *C. pacificum* MW861717, and *C. goreaui* KF572161.1) with bwa-mem (v2.2.1)^90^. Full-length reads aligned with more than 90% of identity were kept. The reads aligned on the psbA^ncr^ reference sequence covered by the highest number of reads were selected to build a consensus sequence for each sample (samtools mpileup). We selected two psbA^ncr^ sequences for each *Cladocopium* clade identified in ref. ^29^ and aligned them with the 82 consensus sequences with clustalW (MegaX software)^91^. A Bayesian phylogeny of this alignment was generated using MrBayes (v3.2.7a)^92^ with the GTR substitution model (nst=6), gamma-distributed rate variation (rates=invgamma), the birth-death clock model (brlenspr=clock:birthdeath). The MCMC was run for 1,000,000 generations with sampling every 1000 steps and the first 25% was removed as burnin. The phylogeny was represented with R packages ape (v5.6.2) and ggtree (v3.6.2)^93^ (Supplementary Fig. 2c).

### Procrustean analysis of Cophylogeny (PACo)

To test for phylogenetic congruence between *Pocillopora* hosts and *Cladocopium* photosymbionts we used the R package paco (v0.4.2) to conduct a Procrustean analysis of cophylogeny (PACo)^94^. The PACo approach uses a distance-based global fit to quantify the topological congruence between two phylogenetic trees and identifies the associations contributing most strongly to the cophylogenetic structure^29^. For *Cladocopium*, we used the SNP frequency clustering described above. For *Pocillopora*, we used a subsetted phylogenetic tree generated from the SVD quartet phylogeny of Hume et al.^27^ generated by selecting a single representative for each of the five host lineages. The host and photosymbiont phylogenetic trees were aligned with the untangle function (step2side method) of the R package dendextend (v1.17.1)^95^.

### Gene expression levels of *Pocillopora* and *Cladocopium* from metatranscriptomic reads

Metatranscriptomic reads (Illumina-generated 150-bp, paired-end) were separately aligned to predicted coding sequences (CDS) of the *Pocillopora meandrina* coral host reference genome (Noel et al.^84^), and Symbiodiniaceae sequences (the CDS of the *Cladocopium goreaui* genome^32^ and a *Durusdinium* transcriptome^96^) using Burrows–Wheeler Transform Aligner (BWA-mem, v0.7.15) with the default settings^90^. Host- and symbiont-mapped reads were then sorted and processed using SAMtools v1.10.2^97^ to generate respective bam files. A read was considered a host contig if its sequence aligned to the *P. meandrina* predicted coding sequence with ≥95% of sequence identity and with ≥50% of the sequence aligned. Reads aligned to *Cladocopium goreaui* coding sequences with a cutoff of ≥98% of sequence identity over ≥80% of the read length were retained as symbiont reads. Reads were further filtered to remove those in which more than 75% of the read length was low complexity or less than 30% was high complexity. Read counts were normalized as transcript per million (TPM). Tables of raw and normalized counts are available^98^.

### Analysis of environment x genotype interactions on gene expression

To evaluate the relative influence of the environment and genotype on the coral host and *Cladocopium* expression profiles we used three complementary approaches, namely (1) quantitative partitioning of gene expression variance into the fraction attributable to each putative driver using a linear mixed model approach (variance partitioning), (2) discriminant analysis of principal components (DAPC) to determine which drivers best distinguish between a priori sample groupings (islands and lineages) based on proportions of correct reassignment, and (3) constrained correspondence analysis informed by both historical and in natura environmental data to identify primary abiotic drivers of gene expression.

#### Quantitative partitioning of gene expression variance

In the first approach, we used the variancePartition package in R (v 1.21.2)^99,100^ to partition the variance attributable to the environment, the host genotype, and the symbiont genotype in the *Pocillopora* and *Cladocopium* expression datasets (35,066 genes for *Pocillopora* and 24,160 genes for *Cladocopium*). Prior to performing the variance partitioning analysis, a correction was applied to remove a bias due to the two different library preparation methods used for RNA sequencing following the procedure described in ref. ^99^. Three variables were defined as random effects: *Pocillopora* genetic lineage, *Cladocopium* genetic lineage, and the island of sampling. In order to account for any potential biases in our estimation of gene expression variance introduced by variancePartition’s random sub-sampling of colonies we repeated the variance partitioning analysis 100 times with a random removal of two samples during each repetition. Genes with a median explained variance >50% for one of the three selected variables across these 100 rounds, hereafter referred to as top variant genes, were then selected for functional enrichment analysis (1821 genes for *Pocillopora* and 2424 genes for *Cladocopium*).

#### Discriminant analysis of principal components

We used the adegenet (v2.1.10) in R^101,102^ to determine which genes are best able to discriminate between pre-defined groups of samples (i.e., based on either their genetic lineage or on their collection environment) and to assess how well these groups could be distinguished from one another based on similarity among their expression profiles. The purpose of DAPC is to find the linear combinations of genes which maximize the differences between pre-specified groups while minimizing the within-group variance thereby allowing for the determination of which genes most strongly discriminate between the pre-defined groups. Coefficients of these genes are called loadings and higher loading scores indicate a stronger discriminatory ability. The linear combinations of these genes’ expression values are referred to as discriminant functions and serve to orient the groups in multi-dimensional space.

We assessed three discriminant analysis clustering models each for the host and algal symbiont with (1) the island of collection (i.e., the environmental effect), (2) coral genetic lineage or photosymbiont genetic lineage as the grouping factor for the coral and photosymbiont, respectively, (i.e., the primary genetic effect), and (3) the genetic lineage of the symbiont (photosymbiont for the host and vice versa; the symbiotic partner effect) as the pre-defined grouping factor. The relative strength of environmental-, genetic-, and symbiont impacts on *Pocillopora* and *Cladocopium* gene expression were then assessed by comparing the proportion of colonies that were correctly re-assigned to their pre-defined groups under each clustering model. A higher proportion of colonies correctly re-assigned to their pre-defined groups indicates a greater power to discriminate between divergent expression profiles and, therefore, a greater influence of that grouping factor on gene expression. The input for these analyses were the normalized expression data (i.e., variance stabilized transformed counts obtained from the package DESeq2, v1.28.1) for all genes with 10 or more counts in at least 90% of colonies with lineage assignations (*n* = 80 colonies; host *n* = 28,972 genes; symbiont *n* = 20,517 genes). We began each model run with an ordination analysis to extract Principal Components (PCs) of gene expression. We then computed a-scores to determine the optimal number of PCs to retain under each clustering model for subsequent cluster identification using discriminant analysis^102^. A-score optimization resulted in our selecting 11 PCs to retain for the host and photosymbiont (for all three models). Group memberships were then independently predicted for the colonies based on DAPC scores.

The number of principal coordinates (PCs) and discriminant functions (DF) retained, the proportion of colonies correctly re-assigned to their a priori groups, and the overall proportion of gene expression variance explained by each factor (sampled island, host lineage, and photosymbiont lineage) are summarized in Supplementary Data 11. We used the proportion of colonies correctly re-assigned to their pre-defined groups as well as loading scores and quantities of top loading genes in order to assess the influence of that grouping factor on gene expression. In addition, we also identified genes whose expression profiles most strongly contributed to that model’s discriminant axes. Genes with discriminant axis loading scores within the upper quartile of scores (i.e., top 25%) were considered as significant contributors to that axis, are referred to hereafter as discriminant genes, and were retained for analysis of functional enrichments. Because there was strong host–symbiont fidelity across all lineages, there was consequently significant overlap between discriminant genes recovered from the primary genetic and symbiotic partner DAPC models. We therefore further divided these discriminant genes into two subcategories: genes which were shared between the two models (shared discriminant genes) and genes which were unique to one of the two models (unique discriminant genes). We use these subcategories to distinguish between analyses of all discriminant genes and unique discriminant genes, respectively, throughout this manuscript.

#### Constrained correspondence analysis of environmental data

We also used an automatic stepwise model-building approach for constrained correspondence analysis (CCA) between the host/symbiont expression data and the environmental data in order to visualize how expression data were structured with respect to potential environmental drivers. To avoid model overfitting we first identified environmental variables that were highly correlated with one another at an *r*^2^ ≥ 0.7 using the corclust function of the klaR package (v1.7.1) in R. From this, we identified clusters of correlated variables (Supplementary Fig. 8) and selected one representative variable from each cluster for further analysis. We then calculated multiple regressions of each environmental variable with the CCA ordination axes of the gene expression data for both the host and photosymbiont separately using the envfit function of the vegan package (v2.5.6). Regression significance was then assessed by permutation tests (10,000 permutations) and top environmental variables were selected as those with environment/expression regression *P* values ≤ 0.05. These environmental variables were then used as the inputs for separate constrained correspondence analyses for the host and photosymbiont using the function cca from the vegan package. Because variance inflation factor analysis of the CCA results indicated the presence of redundant environmental variables, further selection was conducted using an automatic stepwise model selection approach with the ordiR2step function in vegan with 1000 permutations. Environmental variables displaying Bonferroni-adjusted environment/expression regression *P* values ≤ 0.05 were retained and the results visualized to display the strength and forcing direction of each top environmental variable.

### Differential expression and functional enrichment analyses

We assessed differential gene expression between *Pocillopora* hosts containing *Cladocopium* and those containing *Durusdinium* in the far Eastern Tropical pacific (Isla de Las Perlas, Coiba, and Malpelo). Analyses were performed separately for each of the three islands and also for all islands together. The input for each analysis were the host expression data for all genes with 10 or more counts in at least 90% of colonies from that island (*n* = 8/9/6 colonies and *n* = 29,968/30,040/28,936 host genes for Las Perlas/Coiba/Malpelo, respectively) or among the three islands together (*n* = 23 colonies and *n* = 32,060 host genes). For each analysis, these gene counts were normalized and transformed using a variance stabilizing transformation in DESeq2 (v1.28.1)^103^. Genes significantly differentially expressed (DEGs) between colonies containing *Cladocopium* and *Durusdinium* were then identified using DESeq2 with the following criteria: absolute log-fold change (LFC) ≥ 2 after application of ashr log fold-change shrinkage^104^ and FDR-adjusted *P* value ≤ 0.05. Because of the uneven sampling of C- and D-containing colonies between islands, the number of DEGs identified in each inter-island pairwise comparison was highly dependent on the number of colonies input in the DESeq2 analysis. To correct for this and allow for quantitative comparison of DEGs between islands we performed multiple differential expression analyses using two, randomly subsampled, colonies (the lowest sample number) for each category (C- vs D-containing) for each island. This resulted in a range of DEGs estimates from which the mean number of DEGs (±SD) were calculated.

Functional annotations *Pocillopora* genes were obtained from the genomic reference provided in ref*.* ^84^. These annotations were obtained using the Interproscan tool for each coding sequence^105,106^. Gene Ontology (GO) annotations were attributed with the Interpro2GO correspondence table (v2020/06/13)^105^. Functional annotations of *Cladocopium goreaui* genes were recovered from the published genome^32^.

Functional enrichment among top variant (variance partitioning) and discriminant (DAPC) genes were analyzed in the host using the goseq (v1.40.0)^107^ package in R. For top variant genes, the number of GO annotations assigned to genes within that interest group was compared to the number of annotations assigned to the rest of the dataset, to evaluate whether any ontologies were more highly represented within the module than expected by chance (i.e., Fisher’s exact test in goseq). *Cladocopium* functional enrichment analyses were performed using independent Fisher’s exact tests for each Pfam (protein family) domain between genes present in each gene-of-interest category (top variant, discriminant, etc.) as compared to all expressed genes of *Cladocopium*. *P* values were corrected for multiple testing with the Benjamini & Hochberg method implemented in the p.adjust function in R. For each Pfam term, the number of annotations assigned to genes within an interest group (i.e., top variant and discriminant genes) was compared to the number of annotations assigned to the genome, to evaluate whether any ontologies were more highly represented within the module than expected by chance (Fisher’s exact test).

### Reporting summary

Further information on research design is available in the Nature Portfolio Reporting Summary linked to this article.

## Supplementary information

Supplementary Information

Description of Additional Supplementary Files

Supplementary Data 1-19

Reporting Summary

## Data Availability

The genomic data used in this study have been deposited in the European Nucleotide Archive database under accession code PRJEB47249. The ITS2 data used in this study have been deposited in the European Nucleotide Archive database under accession code PRJEB52458. The metatranscriptomic data generated in this study have been deposited in the European Nucleotide Archive database under accession code PRJEB52301. The metagenomic used generated in this study have been deposited in the European Nucleotide Archive database under accession code PRJEB52368. The read count and *Cladocopium* metaT-derived filtered SNP data generated in this study have been deposited in a Zenodo repository accessible at: 10.5281/zenodo.7398767^98^. Several, publicly available genomic resources were used for mapping purposes in this study including an Enterobacteria phage PhiX174 genome (GenBank accession code NC_001422.1) and three psbAncr sequences (*C. latusorum* MW819767.1, *C. pacificum* MW861717, and *C. goreaui* KF572161.1). NOAA OI SST V2 High-Resolution Sea Surface Temperature (SST) data were provided by the NOAA PSL, Boulder, Colorado, USA, from their website at https://psl.noaa.gov. Additional source data for the figures presented in this paper are provided alongside custom analysis scripts at the following GitHub repository: https://github.com/institut-de-genomique/TaraPacific_Pocillopora-transcriptomic^108^. All other data are provided as Supplementary Data. Source data are provided with this paper.

## Source data

Source Data

## Code availability

Code for the bioinformatics tools and data workflows is provided at: https://github.com/institut-de-genomique/TaraPacific_Pocillopora-transcriptomic^108^.
