# Supplementary material for: Host transcriptomic plasticity and photosymbiotic fidelity underpin Pocillopora acclimatization across thermal regimes in the Pacific Ocean
Source: Nat Commun. 2023 Jun 1;14:3056. doi: 10.1038/s41467-023-38610-6 (PMC10235041; doi:10.1038/s41467-023-38610-6)
Supplement: Supplementary file 1 — Supplementary Information [file 41467_2023_38610_MOESM1_ESM.pdf]

SUPPLEMENTARY MATERIALS

Supplementary Results.

1. Discriminant analysis of principal components (DAPC)

Under the primary genetic model, *Pocillopora* cf. *effusa* expression profiles are distinguished from all others along the first discriminant function (DF1, Supplementary Fig. 6b). Genes contributing to this axis were enriched in biological processes related to carbohydrate transport (14 genes), lipid biosynthesis (5 genes), immune response (8 genes), tumor necrosis factor-mediated signaling (3 genes), and regulation of response to virus (3 genes; Supplementary Fig. 6c). The second discriminant function (DF2) served to separate the remaining four host lineages from one another, with *P. meandrina* clustered near *P. grandis* and *P. verrucosa* clustered near *SSH5_pver* colonies (Supplementary Fig. 6b). Host genes along DF2 were enriched in processes related to cytolysis in another organism involved in symbiotic interaction (3 genes), ion transport (90 genes), and protein glycosylation and phosphorylation (26 and 197 genes, respectively; Supplementary Fig. 6d and Supplementary Table 5). Functional enrichments shared across both axes included processes related to calcium ion and xenobiotic transport (9/12/16 and 14/16/15 DF1/DF2/shared genes, respectively), lipid biosynthesis and transport (1/1/4 and 6/2/8 genes, respectively), and regulation of apoptosis (28/31/16 genes). Host genes which were unique to the primary genetic lineage model (i.e., not shared with the symbiotic partner model) were principally involved in cytokinesis cell division. These genes were enriched in processes related to centriole replication (1 gene), positive regulation of cytokinesis (1 gene), DNA replication (1 gene), and sphingolipid metabolism (1 gene) along DF1 and processes related to mitosis and meiosis (2 and 1 gene, respectively) and glycerol−3−phosphate metabolism (1 gene) along DF2.

In *Cladocopium*, DF1 under the coral host model distinguished expression profiles from photosymbionts inhabiting *P.* cf. *effusa* corals from all others (Supplementary Fig. 5c) with an enrichment of light-harvesting genes (37 genes) and ion transporters (104 genes; Supplementary Table 6). DF2 distinguished photosymbionts inhabiting *P. meandrina* and *P. grandis* colonies from those inhabiting *P. verrucosa* and *SSH5_pver* colonies with an enrichment of ion transporters (113 genes), and P-loop containing dynein motor regions (13 genes; Supplementary Fig. 5c and Supplementary Table 6).

Finally, under the symbiotic partner model, gene expression profiles of host colonies containing *C. latusorum* L2 symbionts were strongly separated from all others along DF1 (Supplementary Fig. 7b) and carry genes enriched in biological processes related to carbohydrate biosynthesis, binding, and transport (8, 15, and 28 genes, respectively), mitochondrial cytochrome c oxidase assembly (3 genes), tumor necrosis factor-mediated signaling (3 genes), and regulation of response to virus (3 genes; Supplementary Fig. 7c and Supplementary Table 7). DF2 primarily distinguished colonies containing *C. goreaui* and *C. latusorum* L3 photosymbionts from those containing *C. pacificum* (L4 and L5; Supplementary Fig. 7b) and was enriched in processes related to cytolysis in another organism involved in symbiotic interaction (4 genes), phospholipid biosynthesis (8 genes), and nucleosome assembly (8 genes) were enriched (Supplementary Fig. 7d and Supplementary Table 7). Functional enrichments shared across both axes included processes related to xenobiotic and calcium ion transmembrane transport (11/20/14 and 12/10/16 DF1/DF2/shared genes, respectively),

carbohydrate transport (4/5/10 genes), immune response (4/2/5 genes), lipid biosynthesis (1/1/4 genes), regulation of apoptosis (26/28/19 genes). Genes unique to this model and which contributed strongly to DF1 were enriched in processes related to the TCA cycle (1 gene), negative regulation of gluconeogenesis (1 gene), positive regulation of apoptosis (1 gene), regulation of autophagy (1 gene), and response to heat (1 gene). Unique genes contributing to DF2 were enriched in processes related to polysaccharide catabolism (1 gene), meiotic chromosome segregation and chromatin assembly/disassembly (1 gene each), base-excision repair (1 gene), and ammonium transport (2 genes)

Cladocopium gene expression profiles under the primary genetic model distinguish *C. goreaui* (L1, the most divergent lineage) and *C. latusorum* L2 from all others along DF1 (Supplementary Fig. 5d). Because *C. goreaui* is restricted to Rapa Nui island, this result is identical to the environmental model. DF2 serves primarily to distinguish *C. latusorum* L2 from all others (Supplementary Fig. 5d) and along this axis, 113 ion transport genes and 13 P-loop containing dynein motor genes are enriched (Supplementary Table 8).

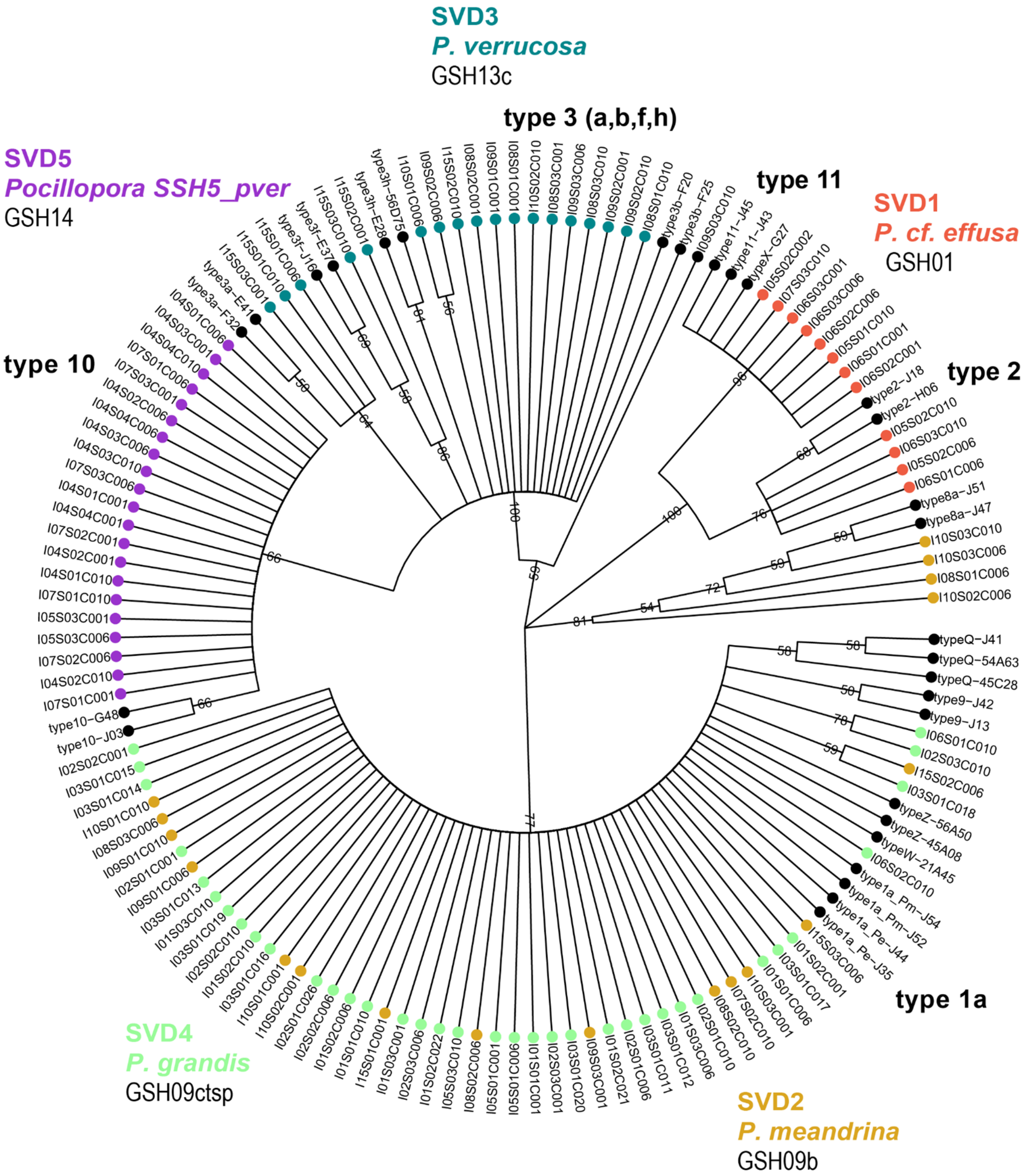

Supplementary Figure 1. Mitochondrial open reading frame (mtORF) based neighbor-joining phylogeny for *Pocillopora*.

Phylogenetic analysis of the mitochondrial open reading frame (mtORF) sequences extracted from metagenomic reads in *Pocillopora* colonies. Colored dots indicate SVD lineages identified in this study. Black dots indicate mtORF sequences of Johnston *et al.* 2022. For each haplotype identified in Johnston *et al.*, two mtORF sequences were randomly chosen. Species hypotheses (GSH) and mtORF types are assigned following Oury *et al.* 2022 and Johnston *et al.* 2022, respectively. Figure originally published in Hume *et al*. and reproduced here with modifications. Source data for this figure are available: https://github.com/institut-de-genomique/TaraPacific_Pocillopora-transcriptomic.

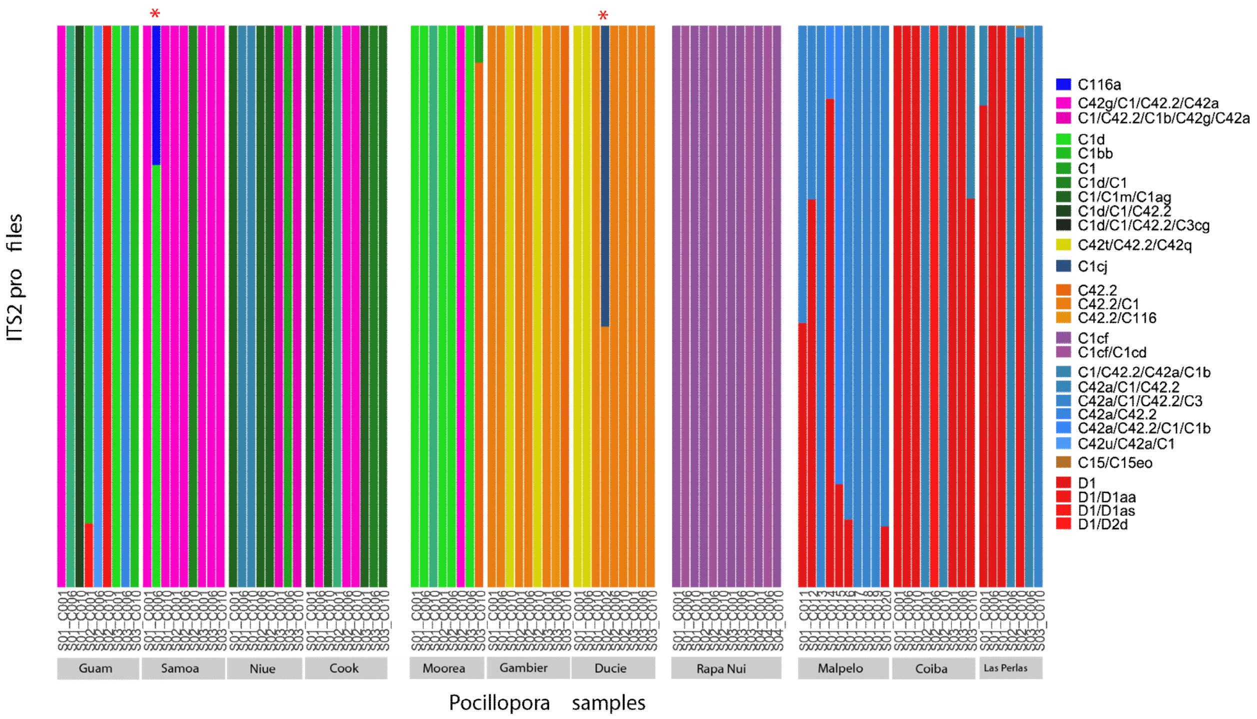

Supplementary Figure 2. Relative abundance of Symbiodiniaceae ITS2 profiles in *Pocillopora* colonies.

Relative abundance of Symbiodiniaceae ITS2 profiles in *Pocillopora* colonies. Each color corresponds to a different profile named by its most abundant ITS2 sequence. *Pocillopora* colonies indicated by an asterisk (top) contain 2 *Cladocopium* ITS2 profiles in large proportion (>25%) and were removed from the population structure analysis. Source data are provided as a Source Data file.

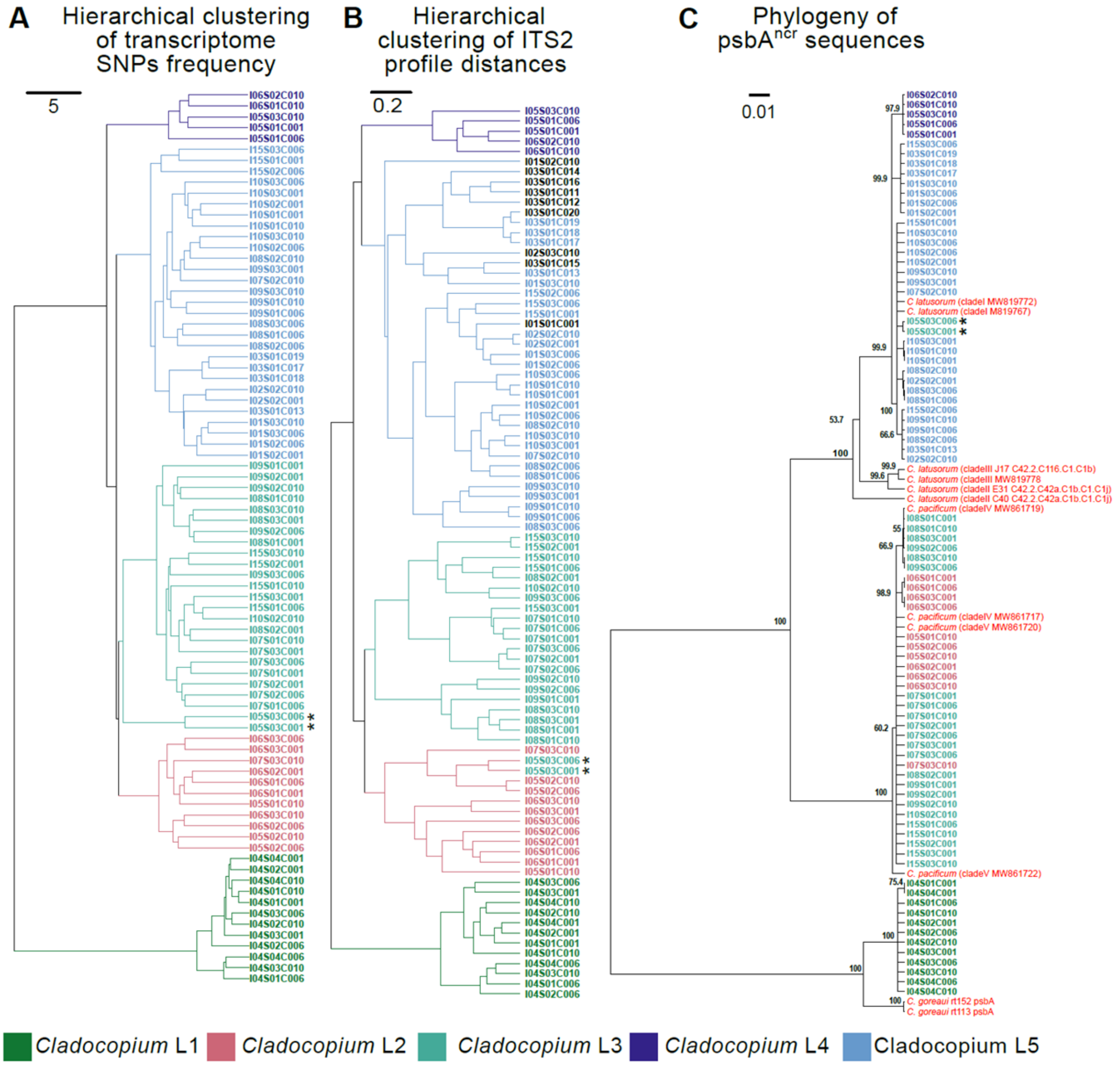

Supplementary Figure 3. Identification of *Cladocopium* lineages in *Pocillopora* coral colonies.

(a) Hierarchical clustering of the frequency of 3,712 SNPs detected by the mapping of metatranscriptomic reads on 1,354 genes of the *C. goreaui* genome. The 5 lineages named *Cladocopium* L1 to L5 correspond to the optimal number of clusters defined with the Gap statistic method. Sample name colors correspond to these 5 lineages. (b) Hierarchical clustering of ITS2 profile BrayCurtis distances between each pair of samples. ITS2 sequences of *Cladocopium*-containing *Pocillopora* colonies were analyzed using SymPortal[80]. (c) Bayesian phylogeny of psbA^{ncr} sequence. Bayesian posterior probabilities are indicated at each node. Red taxa are from Turnham *et al.* 2021[31], Johnston *et al.* 2022[32] for *C. latusorum*/*C. pacificum* and Thornhill *et al.* 2014[36] for *C. goreaui*. Black stars point out the two *Cladocopium* lineages with incoherent positioning between the 3 trees. Source data for this figure are available: https://github.com/institut-de-genomique/TaraPacific_Pocillopora-transcriptomic.

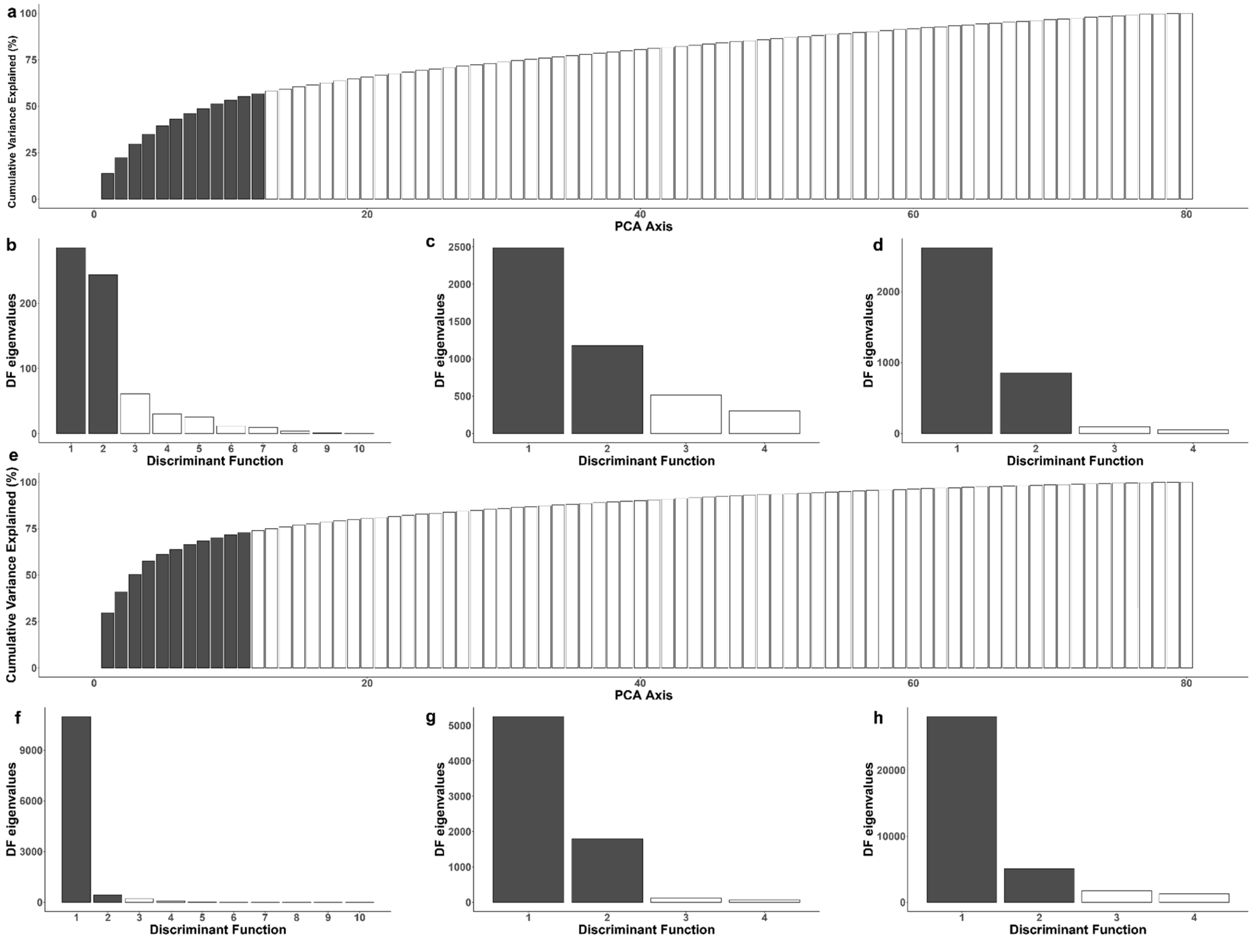

Supplemental Figure 4. DAPC Eigenvalues.

Cumulative percentage of variance explained by the principal coordinate axes (PCA) of the environmental DAPC model for the host (a) and photosymbiont (e). Shaded bars indicate the PCAs retained in each model respectively. Discriminant function eigenvalues for the DAPC models grouped by the environment (b, f), the primary lineage (c, g), and the lineage of the symbiotic partner (d, h) for the host and symbiont, respectively. Source data are provided as a Source Data file.

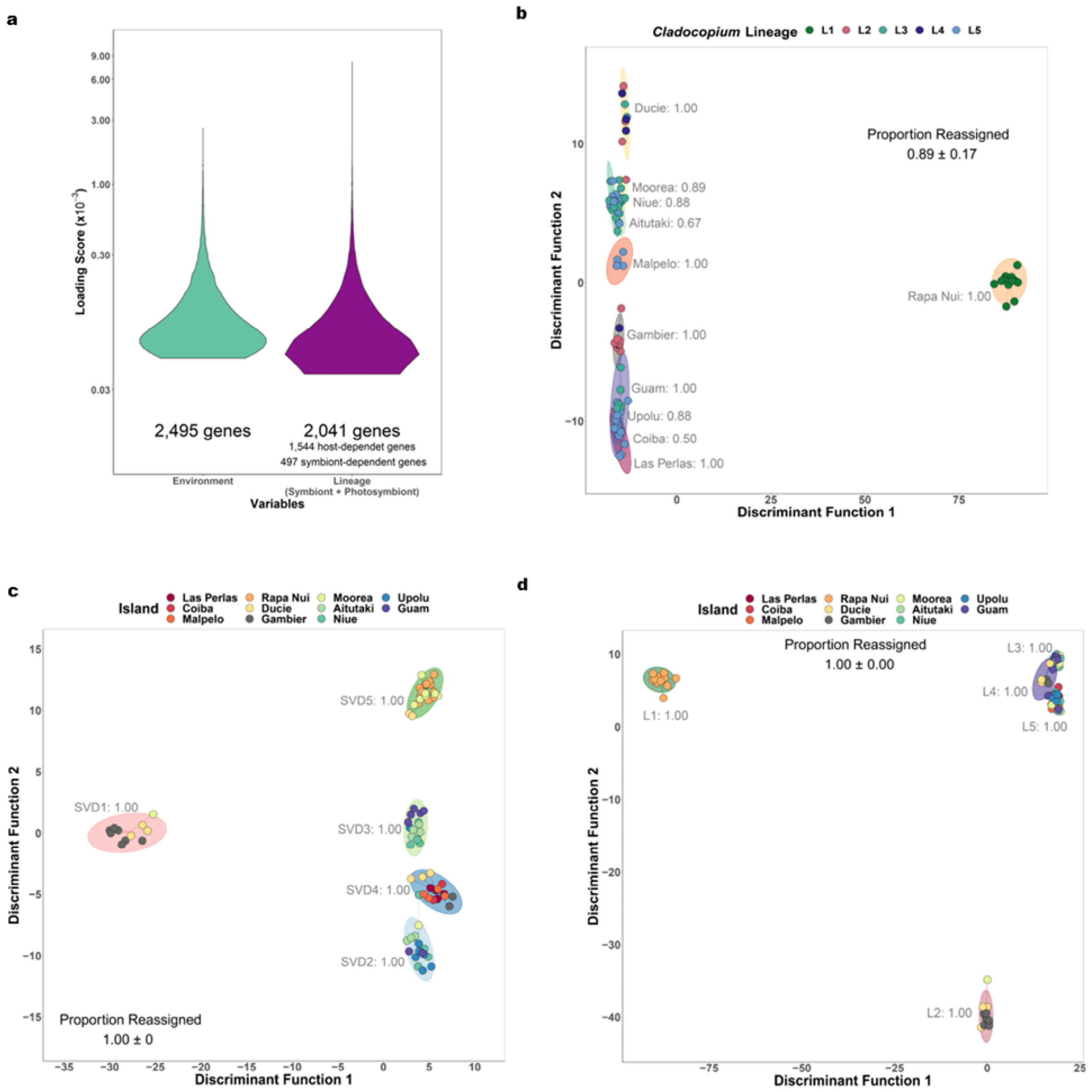

Supplementary Figure 5. Discriminant analysis of principal components (DAPC) of *Cladocopium* symbiont gene expression data.

(a) Loading scores of top discriminant genes revealed a stronger influence of genetic lineage than of the environment in *Cladocopium*. DAPC scatter plots show expression profiles for the *Cladocopium* photosymbiont when colonies were grouped by (b) their island of collection, (c) host lineage, and (d) photosymbiont lineage. Points represent individual colony expression profiles and are colored by photosymbiont lineage (panel b) or island of sampling (panels c and d). Shaded ellipses denote 95%-confidence intervals around the group mean. Group-specific proportions of correct reassignments are indicated within each cluster (labels) and overall model proportion of correct reassignment (mean ± standard deviation) are also presented. Source data are provided as a Source Data file.

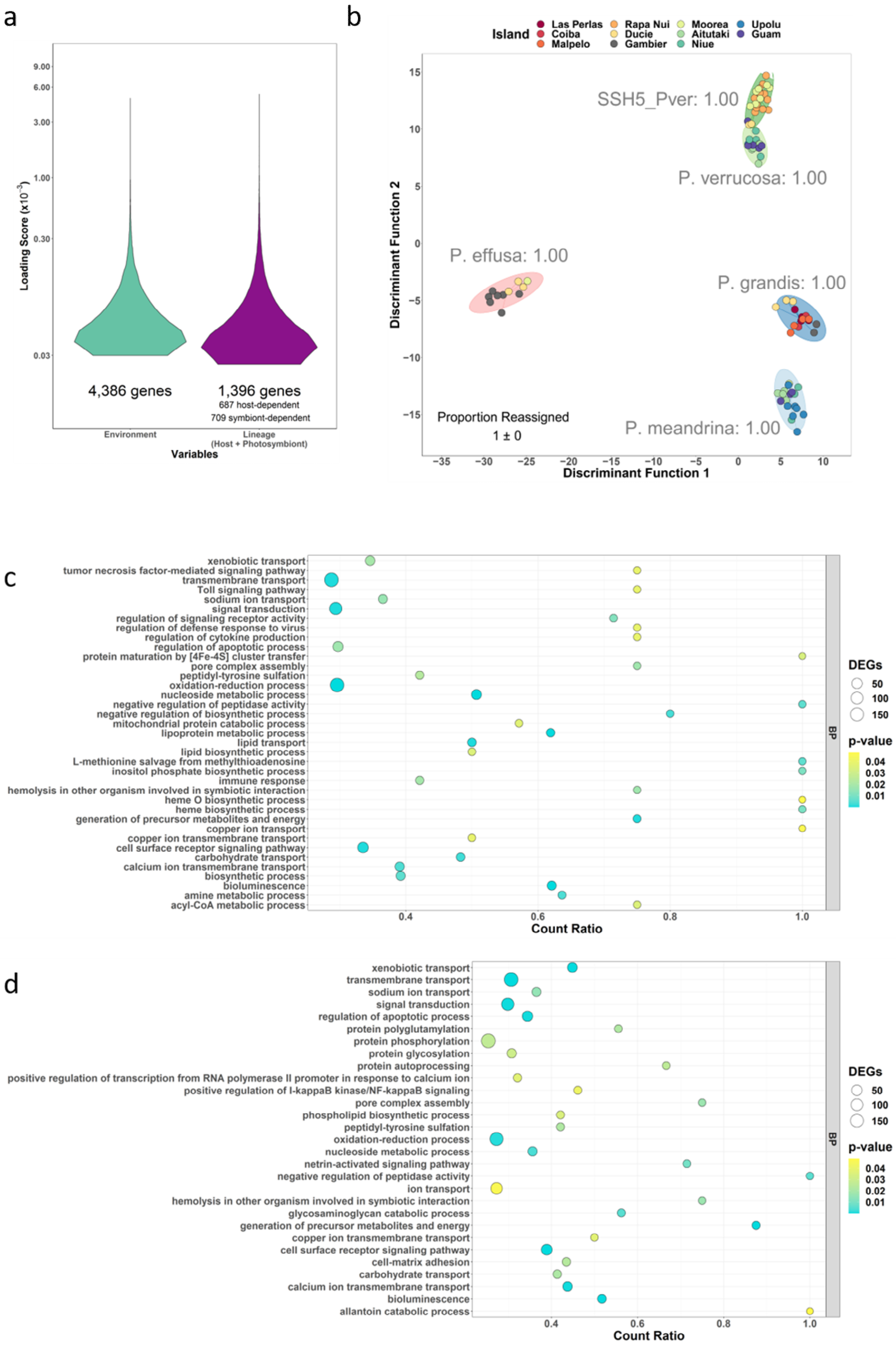
a
b
c
d
Loading Score (x10^-3)
9.00
6.00
3.00
1.00
0.30
0.03
4,386 genes
1,396 genes
687 host-dependent
709 symbiont-dependent
Environment
Lineage
(Host + Photosymbiont)
Variables
Island
Las Perlas
Coiba
Malpelo
Rapa Nui
Ducie
Gambier
Moorea
Aitutaki
Niue
Upolu
Guam
SSH5_Pver: 1.00
P. verrucosa: 1.00
P. effusa: 1.00
P. grandis: 1.00
P. meandrina: 1.00
Proportion Reassigned
1 ± 0
Discriminant Function 1
Discriminant Function 2
xenobiotic transport
tumor necrosis factor-mediated signaling pathway
transmembrane transport
Toll signaling pathway
sodium ion transport
signal transduction
regulation of signaling receptor activity
regulation of defense response to virus
regulation of cytokine production
regulation of apoptotic process
protein maturation by [4Fe-4S] cluster transfer
pore complex assembly
peptidyl-tyrosine sulfation
oxidation-reduction process
nucleoside metabolic process
negative regulation of peptidase activity
negative regulation of biosynthetic process
mitochondrial protein catabolic process
lipoprotein metabolic process
lipid transport
lipid biosynthetic process
L-methionine salvage from methylthioadenosine
inositol phosphate biosynthetic process
immune response
hemolysis in other organism involved in symbiotic interaction
heme O biosynthetic process
heme biosynthetic process
generation of precursor metabolites and energy
copper ion transport
copper ion transmembrane transport
cell surface receptor signaling pathway
carbohydrate transport
calcium ion transmembrane transport
biosynthetic process
bioluminescence
amine metabolic process
acyl-CoA metabolic process
Count Ratio
BP
DEGs
50
100
150
p-value
0.04
0.03
0.02
0.01
xenobiotic transport
transmembrane transport
sodium ion transport
signal transduction
regulation of apoptotic process
protein polyglutamylation
protein phosphorylation
protein glycosylation
protein autoprocessing
positive regulation of transcription from RNA polymerase II promoter in response to calcium ion
positive regulation of I-kappaB kinase/NF-kappaB signaling
pore complex assembly
phospholipid biosynthetic process
peptidyl-tyrosine sulfation
oxidation-reduction process
nucleoside metabolic process
netrin-activated signaling pathway
negative regulation of peptidase activity
ion transport
hemolysis in other organism involved in symbiotic interaction
glycosaminoglycan catabolic process
generation of precursor metabolites and energy
copper ion transmembrane transport
cell surface receptor signaling pathway
cell-matrix adhesion
carbohydrate transport
calcium ion transmembrane transport
bioluminescence
allantoin catabolic process
Count Ratio
BP
DEGs
50
100
150
p-value
0.04
0.03
0.02
0.01

Supplementary Figure 6. Discriminant analysis of principal components (DAPC) of *Pocillopora* host gene expression data grouped by the host genetic lineage and biological process functional enrichments of top discriminant genes.
(a) Loading scores of top discriminant genes revealed a roughly equivalent influence of the environment and of genetic lineage on gene expression in the *Pocillopora* host. (b) DAPC scatter plot showing expression profiles for the *Pocillopora* host when colonies were grouped by their respective genetic lineage. Points represent individual colony expression profiles and are colored by island of sampling. Shaded ellipses denote 95%-confidence intervals around the group (SVD clade) mean. Group-specific proportions of correct reassignments are indicated within each cluster (labels) and overall model proportion of correct reassignment (mean ± standard deviation) is presented in the bottom left. Gene Ontology enrichment analysis dot plots showing the top enriched biological process GO terms identified for each gene from comparison to a Wallenius noncentral hypergeometric sampling distribution allowing for p-value calculation after accounting for selection bias and correction for multiple testing (FDR ≤ 0.05) using GOSeq (v1.40.0) from among *Pocillopora* host genes contributing most strongly to (c) the first and (d) the second discriminant function. The size of each dot reflects the ratio of enriched genes to total genes sharing that GO term, and the color of the dot indicates the enrichment significance. Source data are provided as a Source Data file.

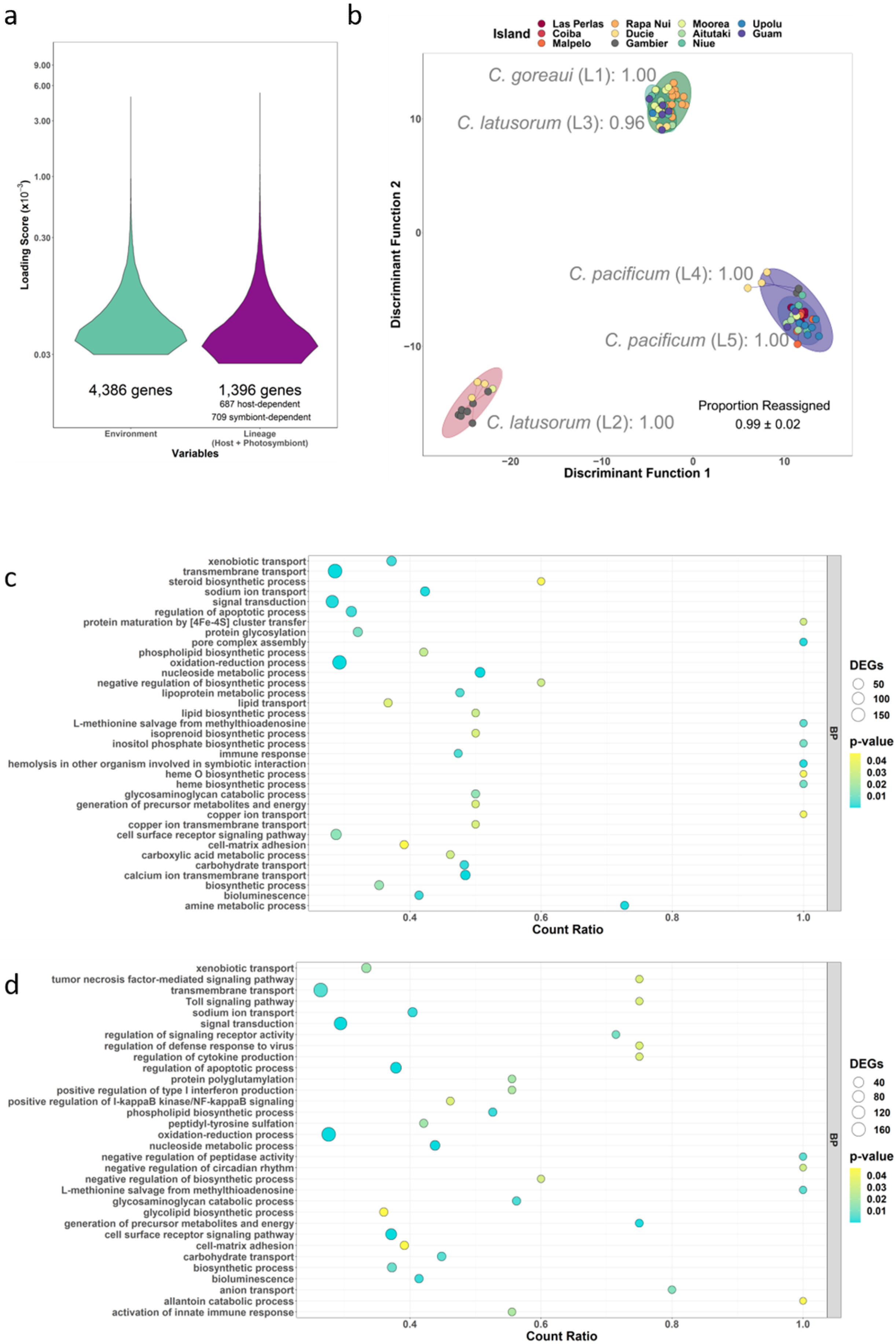
a
Loading Score (x10^-3)
9.00
6.00
3.00
1.00
0.30
0.03
4,386 genes
1,396 genes
687 host-dependent
709 symbiont-dependent
Environment
Lineage
(Host + Photosymbiont)
Variables
b
Island
Las Perlas
Coiba
Malpelo
Rapa Nui
Ducie
Gambier
Moorea
Aitutaki
Niue
Upolu
Guam
C. goreaui (L1): 1.00
C. latusorum (L3): 0.96
C. pacificum (L4): 1.00
C. pacificum (L5): 1.00
C. latusorum (L2): 1.00
Proportion Reassigned
0.99 ± 0.02
Discriminant Function 2
Discriminant Function 1
c
xenobiotic transport
transmembrane transport
steroid biosynthetic process
sodium ion transport
signal transduction
regulation of apoptotic process
protein maturation by [4Fe-4S] cluster transfer
protein glycosylation
pore complex assembly
phospholipid biosynthetic process
oxidation-reduction process
nucleoside metabolic process
negative regulation of biosynthetic process
lipoprotein metabolic process
lipid transport
lipid biosynthetic process
L-methionine salvage from methylthioadenosine
isoprenoid biosynthetic process
inositol phosphate biosynthetic process
immune response
hemolysis in other organism involved in symbiotic interaction
heme O biosynthetic process
heme biosynthetic process
glycosaminoglycan catabolic process
generation of precursor metabolites and energy
copper ion transport
copper ion transmembrane transport
cell surface receptor signaling pathway
cell-matrix adhesion
carboxylic acid metabolic process
carbohydrate transport
calcium ion transmembrane transport
biosynthetic process
bioluminescence
amine metabolic process
Count Ratio
BP
DEGs
50
100
150
p-value
0.04
0.03
0.02
0.01
d
xenobiotic transport
tumor necrosis factor-mediated signaling pathway
transmembrane transport
Toll signaling pathway
sodium ion transport
signal transduction
regulation of signaling receptor activity
regulation of defense response to virus
regulation of cytokine production
regulation of apoptotic process
protein polyglutamylation
positive regulation of type I interferon production
positive regulation of I-kappaB kinase/NF-kappaB signaling
phospholipid biosynthetic process
peptidyl-tyrosine sulfation
oxidation-reduction process
nucleoside metabolic process
negative regulation of peptidase activity
negative regulation of circadian rhythm
negative regulation of biosynthetic process
L-methionine salvage from methylthioadenosine
glycosaminoglycan catabolic process
glycolipid biosynthetic process
generation of precursor metabolites and energy
cell surface receptor signaling pathway
cell-matrix adhesion
carbohydrate transport
biosynthetic process
bioluminescence
anion transport
allantoin catabolic process
activation of innate immune response
Count Ratio
BP
DEGs
40
80
120
160
p-value
0.04
0.03
0.02
0.01

Supplementary Figure 7. *Discriminant analysis of principal components (DAPC) of Pocillopora host gene expression data grouped by the photosymbiont genetic lineage and biological process functional enrichments of top discriminant genes.*

(a) Loading scores of top discriminant genes revealed a roughly equivalent influence of the environment and of genetic lineage on gene expression in the *Pocillopora* host. (b) DAPC scatter plot showing expression profiles for the *Pocillopora* host when colonies were grouped by their dominant photosymbiont's genetic lineage. Points represent individual colony expression profiles and are colored by island of sampling. Shaded ellipses denote 95%-confidence intervals around the group (*Cladocopium C1/C42* lineage) mean. Group-specific proportions of correct reassignments are indicated within each cluster (labels) and overall model proportion of correct reassignment (mean ± standard deviation) is presented in the upper right. Gene Ontology enrichment analysis dot plots showing the top enriched biological process GO terms identified for each gene from comparison to a Wallenius noncentral hypergeometric sampling distribution allowing for p-value calculation after accounting for selection bias and correction for multiple testing (FDR ≤ 0.05) using GOSeq (v1.40.0) from among *Pocillopora* host genes contributing most strongly to (c) the first and (d) the second discriminant function. The size of each dot reflects the ratio of enriched genes to total genes sharing that GO term, and the color of the dot indicates the enrichment significance. Source data are provided as a Source Data file.

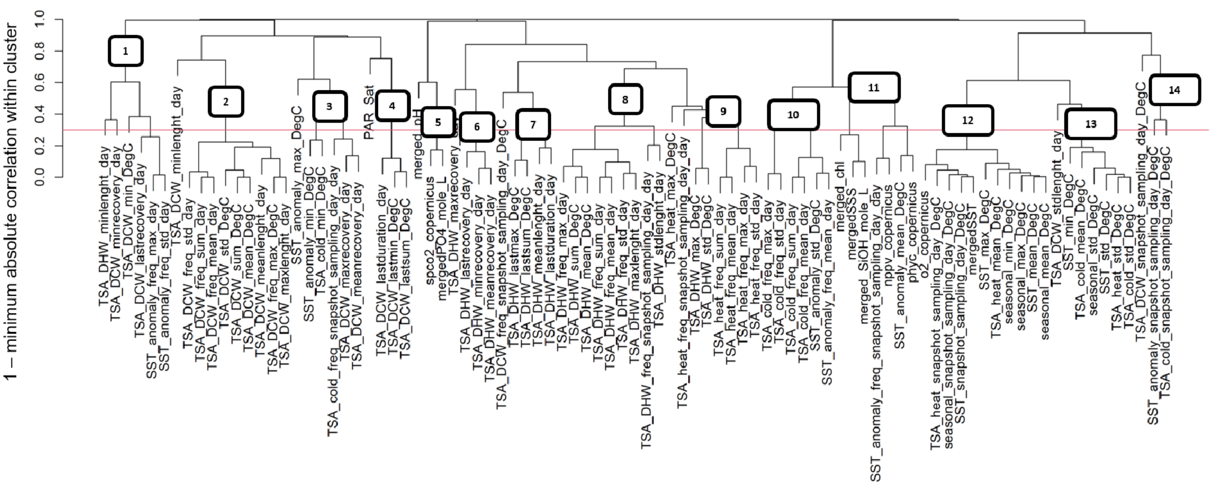

Supplementary Figure 8. *Clustering of environmental variables and identification of correlation clusters.*

Hierarchical clustering of the absolute correlations between environmental variables. Grouped branches extending below the red line demarcate highly correlated variable clusters ($r^2 \geq 0.7$) and are given numerical identifiers. Source data for this figure are available in supplementary data 13. Summary of acronyms, and data collection methods available here: https://zenodo.org/record/6499374/files/README_TaraPacific_historical_SST.md.

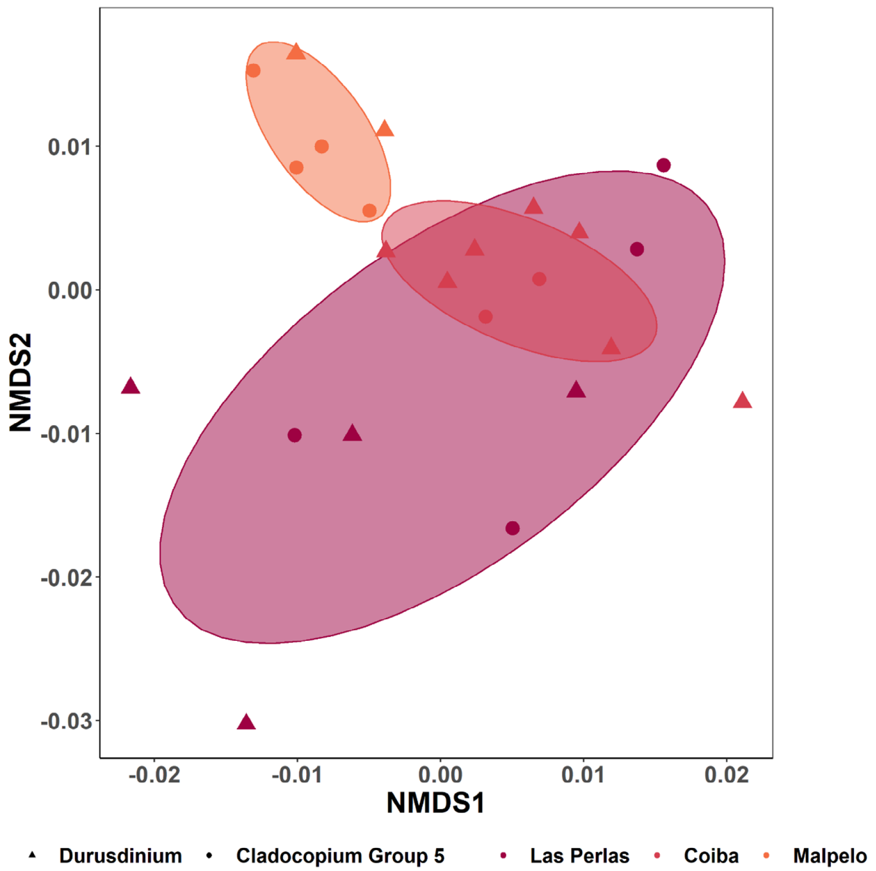

Supplementary Figure 9. *MDS plot of global host gene expression profiles of Cladocopium- and Durusdinium-containing Pocillopora SVD4 colonies in the Eastern Tropical Pacific.* Data are colored by island (Las Perlas, Coiba, and Malpelo) with shapes denoting the dominant symbiont community member (*Durusdinium D1* - triangle, *C. latusorum* L5 - circle). Shaded ellipses denote 95%-confidence intervals around the group (island) mean. Expression profile dispersions were not significantly explained by symbiont community member (PERMANOVA, *P* > 0.05). Source data are provided as a Source Data file.

a

b

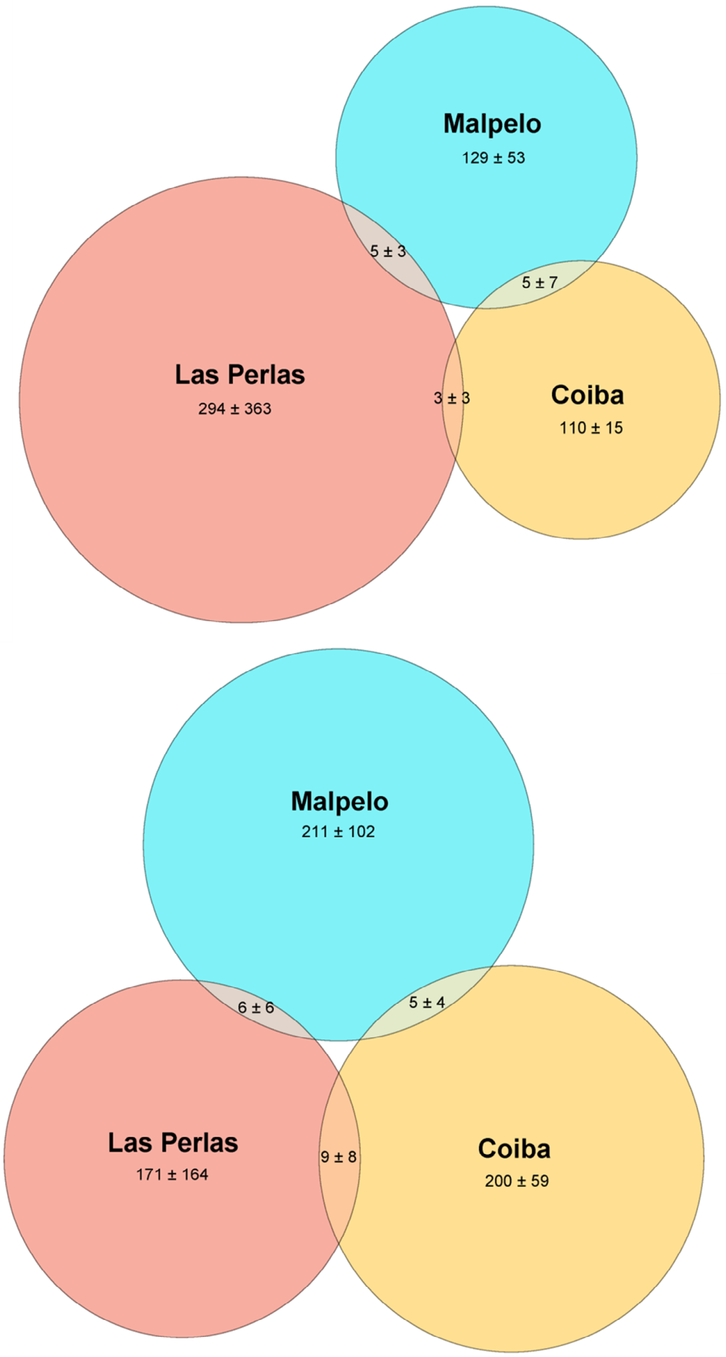

Supplementary Figure 10. *Summary of host genes differentially expressed between C. latusorum- and Durusdinium-containing Pocillopora SVD4 colonies in the Eastern Tropical Pacific.*

Mean number (± sd) of *Pocillopora* host genes. (a) Up- and (b) down-regulated at |LFC| ≥ 2 and two-tailed Wald test FDR-adjusted $P \leq 0.05$ in *Durusdinium*-containing colonies relative to those with *Cladocopium* after controlling for discrepant colony numbers on three islands in the Eastern Tropical Pacific (Isla de las Perlas, Coiba, and Malpelo). Source data are provided as a Source Data file.
