# Supplementary material for: Host transcriptomic plasticity and photosymbiotic fidelity underpin Pocillopora acclimatization across thermal regimes in the Pacific Ocean
Source: Nat Commun. 2023 Jun 1;14:3056. doi: 10.1038/s41467-023-38610-6 (PMC10235041; doi:10.1038/s41467-023-38610-6)
Supplement: Supplementary file 2 — Description of Additional Supplementary Files [file 41467_2023_38610_MOESM2_ESM.pdf]

Description of Additional Supplementary Files

File Name: Supplementary Data 1
Description: Gene IDs and interproscan-derived functional annotations for the *Pocillopora* host genes identified through variation partitioning analysis as having greater than 50% of their variation in expression (i.e., top variant genes) explained by the sampling environment (Environment), the host genetic lineage (Host Lineage), or the photosymbiont genotype (Photosymbiont Lineage). Gene ID: gene identifier; Variable: the factor to which expression variation is linked; VarianceMedian: median proportion of expression variation explained; sd: standard deviation of the proportion of expression variation explained; Q1: first quartile of proportion of expression variation explained; Q3: third quartile of proportion of expression variation explained; IPR Term: introproscan-derived functional term; IPR Name: introproscan-derived functional annotation

File Name: Supplementary Data 2
Description: Gene IDs and protein family-derived functional annotations for the *Cladocopium* photosymbiont genes identified through variation partitioning analysis as having greater than 50% of their variation in expression explained by the sampling environment (Environment), the host genetic lineage (Host Lineage), or the photosymbiont genotype (Photosymbiont Lineage). Gene ID: gene identifier; Variable: the factor to which expression variation is linked; VarianceMedian: median proportion of expression variation explained; sd: standard deviation of the proportion of expression variation explained; Q1: first quartile of proportion of expression variation explained; Q3: third quartile of proportion of expression variation explained; PFAM1: protein-family derived term; PFAM1.Sum: protein-family derived term summary; PFAM1.Def: protein family-derived functional annotation.

File Name: Supplementary Data 3
Description: Summary of biological process gene ontology functional enrichments among environment-associated top variant and discriminant genes in the *Pocillopora* host. Analysis: the analysis from which the genes were derived - either variation partitioning (varPart) or discriminant analysis of principal components (DPAC); Variable: the factor or discriminant axis (DF1 or DF2) to which expression variation is linked; Gene Ontology Category: gene ontology category; Over Represented p-value: Wallenius noncentral hypergeometric sampling distribution-derived p-value for the associated GO category being over represented amongst differentially expressed genes; Under Represented p-value: Wallenius noncentral hypergeometric sampling distribution-derived p-value for the associated GO category being over represented amongst differentially expressed genes; Number of differentially expressed genes in Category: the number of differentially expressed genes in the associated GO category; Number of genes in Category: the total number of genes in the associated GO category; GO Term: the gene ontology term; Gene Ontology: one of either biological process (BP), molecular function (MF), or cellular compartment (CC); Over Represented FDR: false discovery rate value for the associated GO category being over represented amongst differentially expressed genes; Full GO Term: the gene ontology and gene ontology term; Gene IDs: gene identifiers for the genes in the category.

File Name: Supplementary Data 4
Description: Protein family functional enrichments among the top variant and discriminant genes linked to the sampling environment identified within the *Cladocopium* photosymbiont. Fisher test, * P ≤ 0.05; ** P ≤ 0.01; *** P ≤ 0.001. Analysis: the analysis from which the genes were derived - either variation partitioning (varPart) or discriminant analysis of principal components (DPAC); Variable: the factor or discriminant axis (DF1 or DF2) to which expression variation is linked; PFAM: the protein family term; Number of enriched genes carrying the Pfam domain: the number of enriched genes with the associated Pfam term; Total number genes carrying the Pfam domain: the total number of gene with the associated Pfam term; pvalue: one-tailed Fisher's test un-adjusted p-value; odds.ratio: one-tailed Fisher's test odds ratio; adjusted p-value: one-tailed Fisher's test p-value adjusted for multiple comparisons; PFAM summary: summary of protein family term; PFAM description: protein family functional description.

File Name: Supplementary Data 5
Description: Summary of biological process gene ontology functional enrichments among host genetic lineage-associated top variant and discriminant genes in the *Pocillopora* host. Analysis: the analysis from which the genes were derived - either variation partitioning (varPart) or discriminant analysis of principal components (DPAC); Variable: the factor or discriminant axis (DF1 or DF2) to which expression variation is linked; Gene Ontology Category: gene ontology category; Over Represented p-value: p-value for the associated GO category being over represented amongst differentially expressed genes; Under Represented p-value: p-value for the associated GO category being over represented amongst differentially expressed genes; Number of differentially expressed genes in Category: the number of differentially expressed genes in the associated GO category; Number of genes in Category: the total number of genes in the associated GO category; GO Term: the gene ontology term; Gene Ontology: one of either biological process (BP), molecular function (MF), or cellular compartment (CC); Over Represented FDR: false discovery rate value for the associated GO category being over represented amongst differentially expressed genes; Full GO Term: the gene ontology and gene ontology term; Gene IDs: gene identifiers for the genes in the category.

File Name: Supplementary Data 6
Description: Protein family functional enrichments among the top variant and discriminant genes linked to the photosymbiont genetic lineage identified within the *Cladocopium* photosymbiont. Fisher test, * P ≤ 0.05; ** P ≤ 0.01; *** P ≤ 0.001. Analysis: the analysis from which the genes were derived - either variation partitioning (varPart) or discriminant analysis of principal components (DPAC); Variable: the factor or discriminant axis (DF1 or DF2) to which expression variation is linked; PFAM: the protein family term; Number of enriched genes carrying the Pfam domain: the number of enriched genes with the associated Pfam term; Total number genes carrying the Pfam domain: the total number of gene with the associated Pfam term; pvalue: one-tailed Fisher's test un-adjusted p-value; odds.ratio: one-tailed Fisher's test odds ratio; adjusted p-value: one-tailed Fisher's test p-value adjusted for multiple comparisons; PFAM summary: summary of protein family term; PFAM description: protein family functional description.

File Name: Supplementary Data 7
Description: Summary of biological process gene ontology functional enrichments among photosymbiont lineage-associated top variant and discriminant genes in the *Pocillopora* host. Analysis: the analysis from which the genes were derived - either variation partitioning (varPart) or discriminant analysis of principal components (DPAC); Variable: the factor or discriminant axis (DF1 or DF2) to which expression variation is linked; Gene Ontology Category: gene ontology category; Over Represented p-value: p-value for the associated GO category being over represented amongst differentially expressed genes; Under Represented p-value: p-value for the associated GO category being over represented amongst differentially expressed genes; Number of differentially expressed genes in Category: the number of differentially expressed genes in the associated GO category; Number of genes in Category: the total number of genes in the associated GO category; GO Term: the gene ontology term; Gene Ontology: one of either biological process (BP), molecular function (MF), or cellular compartment (CC); Over Represented FDR: false discovery rate value for the associated GO category being over represented amongst differentially expressed genes; Full GO Term: the gene ontology and gene ontology term; Gene IDs: gene identifiers for the genes in the category.

File Name: Supplementary Data 8
Description: Protein family functional enrichments among the top variant and discriminant genes linked to the host genetic lineage identified within the *Cladocopium* photosymbiont. Fisher test, * $P \leq 0.05$; ** $P \leq 0.01$; *** $P \leq 0.001$. Analysis: the analysis from which the genes were derived - either variation partitioning (varPart) or discriminant analysis of principal components (DPAC); Variable: the factor or discriminant axis (DF1 or DF2) to which expression variation is linked; PFAM: the protein family term; Number of enriched genes carrying the Pfam domain: the number of enriched genes with the associated Pfam term; Total number genes carrying the Pfam domain: the total number of gene with the associated Pfam term; pvalue: one-tailed Fisher's test un-adjusted p-value; odds.ratio: one-tailed Fisher's test odds ratio; adjusted p-value: one-tailed Fisher's test p-value adjusted for multiple comparisons; PFAM summary: summary of protein family term; PFAM description: protein family functional description.

File Name: Supplementary Data 9
Description: Model fit results for the six discriminant analysis of principal component (DAPC) models. PCs/DFs Retained: the number of principal components and discriminant functions retained; Var Explained: the proportion of expression variation explained by the model; Prop Reassigned: the proportion of colonies correctly re-assigned to their *a priori* groups for each model.

File Name: Supplementary Data 10
Description: Permutational multivariate analysis of variance (PERMANOVA) model information. Table containing the results of permutational multivariate analysis of variance (PERMANOVA) for *Pocillopora* and *Cladocopium* gene expression under the three *a priori* single factor grouping scenarios (environment, primary lineage, and symbiotic partner) as well as for the interactive model (environment *x* primary genetic lineage). DF: degrees of freedom; Sum Sq: sum of

squares; Mean Sq: mean sum of squares; Model F: F value; R2: model r^2 value; PVAL: PERMANOVA p-values, P ≤ 0.05 *; P ≤ 0.01 **; P ≤ 0.001 ****.

File Name: Supplementary Data 11
Description: Permutational fitting of environmental variables to gene expression data.
Table containing the results of permutational fitting of environmental variables to the gene expression data (vegan::envfit) in *Pocillopora* and *Cladocopium*. Top contributing variables were defined as having $P \leq 0.05$ and $P \leq 0.001$ for the host and photosymbiont, respectively. Cumulative R2 adj: cumulative model adjusted r^2; DF: degrees of freedom; AIC: *Akaike* information criterion; F: model F-value; P.ADJ: ordiR2step permutational *ANOVA* adjusted p-value.

File Name: Supplementary Data 12
Description: Summary table of *Pocillopora* host genes that were significantly differentially expressed (two-tailed Wald test FDR-adjusted $P \leq 0.05$ and $|LFC| \geq 2$) between *Cladocopium*- and *Durusdinium*-containing colonies in all intra- and inter-island comparisons in the Eastern Tropical Pacific. Contrast: the set of islands for which contrasts were examined; No. Colonies: the number of *Cladocopium-* and *Durusdinium*-containing coral colonies in each analysis; Annotated DEG Table: link to the corresponding results table containing functional annotations for significantly differentially expressed genes; Category: whether genes are up- or down-regulated in Durusdinium-containing colonies; DEGs (N): the number of significantly differentially expressed genes; Enriched BP Terms (GO): enriched biological process GO terms associated with the set of significantly differentially expressed genes.

File Name: Supplementary Data 13
Description: Reef site sampling and environmental context data. Historical and in situ environmental data for the islands discussed in this study were selected from the full TARA Pacific data inventory available here: https://doi.org/10.5281/zenodo.6499374. Summary of acronyms and data collection methods available here:
https://zenodo.org/record/6499374/files/README_TaraPacific_historical_SST.md.

File Name: Supplementary Data 14
Description: RNA readset and mapping information. Sample Name: coral colony ID; Tara Sample Name: corresponding Tara Pacific sample ID; Extraction Protocol: the protocol used for RNA extraction; cDNA synthesis protocol: The protocol of cDNA synthesis was either NebNext Ultra II RNA-seq oligo dT (Bioo Scientific, Austin, TX, USA) or TruSeq Stranded mRNA (Illumina, San Diego, CA, USA) ; RNA extraction RIN: RNA integrity score; No. of Valid Sequences: number of valid RNA sequences; Filtered mapped reads on Pocillopora: number of reads mapped to the host genomic reference after filtration; Filtered mapped reads on Symbiodiniaceae: number of reads mapped to the host genomic reference after filtration.

File Name: Supplementary Data 15
Description: DNA readset and mapping information. coral colony ID; Tara Sample Name: corresponding Tara Pacific sample ID; Extraction Protocol: the protocol used for RNA

extraction; No. of Valid Sequences: number of valid RNA sequences; Filtered mapped reads on Pocillopora: number of reads mapped to the host genomic reference after filtration; Filtered mapped reads on Symbiodiniaceae: number of reads mapped to the host genomic reference after filtration.

File Name: Supplementary Data 16
Description: List of genes significantly differentially expressed between *Cladocopium*- and *Durusdinium*-containing *Pocillopora* colonies from Isla de las Perlas and their functional annotations. Significantly up- and down-regulated genes are highlighted in red and blue, respectively. Gene ID: gene name; baseMean: mean of the normalized count values, divided by size factors, taken over all samples; log2FoldChange: the log2-fold change effect size estimate; lfcSE: The standard error estimate for the log2-fold change estimate; pvalue: two-tailed Wald test p-value; padj: adjusted p-value; IPR.Term: interproscan-derived functional annotation; Pfam.Term: protein family-derived functional annotation; GO.Term: gene ontology-derived function annotation.

File Name: Supplementary Data 17
Description: List of genes significantly differentially expressed between *Cladocopium*- and *Durusdinium*-containing *Pocillopora* colonies from Cobia and their functional annotations. Significantly up- and down-regulated genes are highlighted in red and blue, respectively. Gene ID: gene name; baseMean: mean of the normalized count values, divided by size factors, taken over all samples; log2FoldChange: the log2-fold change effect size estimate; lfcSE: The standard error estimate for the log2-fold change estimate; pvalue: two-tailed Wald test p-value; padj: adjusted p-value; IPR.Term: interproscan-derived functional annotation; Pfam.Term: protein family-derived functional annotation; GO.Term: gene ontology-derived function annotation.

File Name: Supplementary Data 18
Description: List of genes significantly differentially expressed between *Cladocopium*- and *Durusdinium*-containing *Pocillopora* colonies from Malpelo and their functional annotations. Significantly up- and down-regulated genes are highlighted in red and blue, respectively. Gene ID: gene name; baseMean: mean of the normalized count values, divided by size factors, taken over all samples; log2FoldChange: the log2-fold change effect size estimate; lfcSE: The standard error estimate for the log2-fold change estimate; pvalue: two-tailed Wald test p-value; padj: adjusted p-value; IPR.Term: interproscan-derived functional annotation; Pfam.Term: protein family-derived functional annotation; GO.Term: gene ontology-derived function annotation.

File Name: Supplementary Data 19
Description: List of genes significantly differentially expressed between *Cladocopium*- and *Durusdinium*-containing *Pocillopora* colonies from all islands in the Eastern Pacific and their functional annotations. Significantly up- and down-regulated genes are highlighted in red and blue, respectively. Gene ID: gene name; baseMean: mean of the normalized count values, divided by size factors, taken over all samples; log2FoldChange: the log2-fold change effect

size estimate; lfcSE: The standard error estimate for the log2-fold change estimate; pvalue: two-tailed Wald test p-value; padj: adjusted p-value; IPR.Term: interproscan-derived functional annotation; Pfam.Term: protein family-derived functional annotation; GO.Term: gene ontology-derived function annotation.
