# Supplementary material for: Host transcriptomic plasticity and photosymbiotic fidelity underpin Pocillopora acclimatization across thermal regimes in the Pacific Ocean
Source: Nat Commun. 2023 Jun 1;14:3056. doi: 10.1038/s41467-023-38610-6 (PMC10235041; doi:10.1038/s41467-023-38610-6)
Supplement: Supplementary file 4 — Reporting Summary [file 41467_2023_38610_MOESM4_ESM.pdf]

nature portfolio

Corresponding author(s): ARMSTRONG, Eric J
CARRADEC, Quentin

Last updated by author(s): May 6, 2023

Reporting Summary

Nature Portfolio wishes to improve the reproducibility of the work that we publish. This form provides structure for consistency and transparency in reporting. For further information on Nature Portfolio policies, see our Editorial Policies and the Editorial Policy Checklist.

Statistics

For all statistical analyses, confirm that the following items are present in the figure legend, table legend, main text, or Methods section.

n/a	Confirmed	
☐	☒	The exact sample size (n) for each experimental group/condition, given as a discrete number and unit of measurement
☐	☒	A statement on whether measurements were taken from distinct samples or whether the same sample was measured repeatedly
☐	☒	The statistical test(s) used AND whether they are one- or two-sided<br>*Only common tests should be described solely by name; describe more complex techniques in the Methods section.*
☐	☒	A description of all covariates tested
☐	☒	A description of any assumptions or corrections, such as tests of normality and adjustment for multiple comparisons
☐	☒	A full description of the statistical parameters including central tendency (e.g. means) or other basic estimates (e.g. regression coefficient) AND variation (e.g. standard deviation) or associated estimates of uncertainty (e.g. confidence intervals)
☐	☒	For null hypothesis testing, the test statistic (e.g. F, t, r) with confidence intervals, effect sizes, degrees of freedom and P value noted<br>*Give P values as exact values whenever suitable.*
☒	☐	For Bayesian analysis, information on the choice of priors and Markov chain Monte Carlo settings
☒	☐	For hierarchical and complex designs, identification of the appropriate level for tests and full reporting of outcomes
☒	☐	Estimates of effect sizes (e.g. Cohen's d, Pearson's r), indicating how they were calculated

Our web collection on statistics for biologists contains articles on many of the points above.

Software and code

Policy information about availability of computer code

Data collection	No software was used for data collection.
Data analysis	Illumina bcl2fastq Conversion software converted raw BCL files generated by RTA to fastq data. For both metagenomic and metatranscriptomic reads we removed short and low-quality nucleotides and adaptor/primer sequences with an in-house script based on Fastx-Toolkit software (https://github.com/institut-de-genomique/fastxtend). For metatranscriptomic reads, read pairs that mapped to ribosomal sequences were removed using the SortMeRNA software (v2.1). We identified a set of genome-wide single nucleotide polymorphisms (SNPs) from metagenomic reads mapped to a Pocillopora meandrina genomic reference using the Genome Analysis Toolkit software (GATK, v3.7.0). Cladocopium SNPs were filtered using VCFtools (v0.1.12) and clustered using the Hclust function of the stats package (v4.2.2) in R (v 4.0.2). The optimal number of clusters was determined using the fviz_nbclust function in the R package factoextra (v1.0.7). Metagenomic reads of the 82 Pocillopora corals containing Cladocopium symbionts were aligned simultaneously on three psbAncr sequences using bwa-mem (v2.2.1). We selected 2 psbAncr sequences for each Cladocopium clade identified in Johnston et al. 2022 and aligned them with the 82 consensus sequences with clustalW (MegaX software). A Bayesian phylogeny of this alignment was generated using MrBayes (v3.2.7a). The phylogeny was represented with R packages ape (v5.6.2) and ggtree (v3.6.2). To test for phylogenetic congruence between Pocillopora hosts and Cladocopium photosymbionts we used the R package paco (v0.4.2). The host and photosymbiont phylogenetic trees were aligned with the untangle function (step2side method) of the R package dendextend (v1.17.1). Metatranscriptomic reads were aligned to predicted coding sequences using BWA-mem (v0.7.15). Mapped reads were then sorted and filtered using SAMtools (v1.10.2). Genotype x Environment interactions on Pocillopora and Cladocopium expression profiles were analyzed using the variancePartition (v 1.21.2), adegenet (v2.1.10), DESeq2 (v1.28.1), klaR (v1.7.1), and Vegan (v 2.5.6) packages from the freely available statistical analysis program, R (v 4.0.2). Gene functional enrichments were analyzed using the goseq (v 1.40.0) package.<br>All custom codes developed in the study are available at the following GitHub repository: https://github.com/institut-de-genomique/TaraPacific_Pocillopora-transcriptomic.

For manuscripts utilizing custom algorithms or software that are central to the research but not yet described in published literature, software must be made available to editors and reviewers. We strongly encourage code deposition in a community repository (e.g. GitHub). See the Nature Portfolio guidelines for submitting code & software for further information.

Data

Policy information about availability of data

All manuscripts must include a data availability statement. This statement should provide the following information, where applicable:

- Accession codes, unique identifiers, or web links for publicly available datasets
- A description of any restrictions on data availability
- For clinical datasets or third party data, please ensure that the statement adheres to our policy

The genomic data used in this study are available under the umbrella project PRJEB47249 [https://www.ebi.ac.uk/ena/browser/view/PRJEB47249] with the ITS2, metatranscriptomic, and metagenomic reads stored within projects PRJEB52458 [https://www.ebi.ac.uk/ena/browser/view/PRJEB52458], PRJEB52301 [https://www.ebi.ac.uk/ena/browser/view/PRJEB52301], and PRJEB52368 [https://www.ebi.ac.uk/ena/browser/view/PRJEB52368], respectively. Read count and Cladocopium metaT-derived filtered SNP data are available in a Zenodo repository: https://doi.org/10.5281/zenodo.7398767. Several, publicly available ,resources were used for mapping purposes in this study including an Enterobacteria phage PhiX174 genome (GenBank: NC_001422.1) and three psbAncr sequences (C. latusorum MW819767.1, C. pacificum MW861717, and C. goreaui KF572161.1). Source data for the figures presented in this paper are provided in the source data file: SourceData.xlsx and/or alongside custom analysis scripts in the following GitHub repository: https://github.com/institut-de-genomique/TaraPacific_Pocillopora-transcriptomic. All other data are available as Supplementary Data within the associated supplementary data file: SupplementaryData_1-19.xlsx.

Field-specific reporting

Please select the one below that is the best fit for your research. If you are not sure, read the appropriate sections before making your selection.

☐ Life sciences ☐ Behavioural & social sciences ☒ Ecological, evolutionary & environmental sciences

For a reference copy of the document with all sections, see nature.com/documents/nr-reporting-summary-flat.pdf

Ecological, evolutionary & environmental sciences study design

All studies must disclose on these points even when the disclosure is negative.

Study description	In this study, we extracted DNA and RNA from A total of 102 Pocillopora spp. colonies from 32 reef sites across 11 islands (Islas de las Perlas, Coïba, Malpelo, Rapa Nui, Ducie, Gambier, Moorea, Aitutaki, Niue, Upolu, and Guam). At each island, fragments of n=3 coral colonies were collected from each of three reef sites yielding a total of ca. n=9 colonies sampled per island (biological replicates).
Research sample	We chose to select Pocillopora meandrina as a study species because of its wide distribution and relatively high abundance within tropical reefs. DNA/RNA from at least 9 individual Pocillopora spp colonies (five lineages including P. meandrina) per reef site and their associated symbiotic dinoflagellate Symbiodiniaceae (five lineages including Cladocopium and Durusdinium) were extracted and served as the raw data in this study. Nine individuals were selected at each site in order to provide as diverse a population as possible (i.e., the highest number of different genets) giving the limitations of storage/sampling cost across the expedition.
Sampling strategy	The Tara Pacific project aimed to deploy the same sampling and analysis protocol at large scale to offer a comparative suite of samples covering the widest environmental envelope while optimizing cruising and sampling time over the 2.5 years of the sampling effort. A set of 11 island systems were targeted to cover the widest possible range of environments in which the study species can be found. At each island, 3 reef sites were selected at which a full sampling was conducted within 4 days. At each site, a total of 3 Pocillopora colonies were selected for sampling. We intended to sample a single species (Pocillopora meandrina) with high replication (n = 9 replicates) at each island in order to adequately quantify differences between genets/genotypes. However, later analysis revealed that at least five species were collected resulting in a reduced n per species at some sites. For each colony fragments were taken for analysis of genomic and transcriptomic data. Each colony was first photographed using a 20 cm quadrat as a scale, their depth recorded, and then sampled to collect about 70 g of each coral by mechanical fragmentation using hammer and chisel. Fragments were placed in Ziploc bags labeled by unique colony ID and brought back to the boat for further processing.
Data collection	Environmental data at the time of sampling was collected via the deployment of a small CTD probe (Castaway CTD) to record in situ temperature and conductivity profiles. Coral depth data was recorded at the time of sampling by dedicated diving teams using a dive slate.
Timing and spatial scale	Sampling of coral colonies occurred over a one year period (2016-2017) as part of the Tara Pacific Expedition.<br>Colonies were sampled from 32 reef sites across 11 islands covering approximately 15,000 km.<br>Sampling at each island was conducted at the following dates:<br>Islas de las Perlas July 17-25 2016, Coïba July 20-23 2016, Malpelo Aug 5 2016, Rapa Nui September 3-7 2016, Ducie September 13-15 2016, September 21-24 2016, Moorea November 6-8 2016, Aitutaki November 14-16 2016, Niue November 22-24 2016, Upolu November 30-December 2 2016, and Guam January 28-30 2017.<br>Pauses in sampling were necessitated by the time required to travel between island groups aboard the Tara sampling vessel.
Data exclusions	No data were excluded from the analyses.
Reproducibility	The transcriptomic data used in this study was independently reanalysed several times using the same research methods and yielded the same results.
Randomization	Coral host samples were assigned to biological groups (i.e., genetic lineages) using a coalescent analysis in RaxML based on a set of curated SNPs identified from mapping host reads to a Pocillopora meandrina reference genome. Symbiont samples were assigned to

biological groups using ITS2 profiles and hierarchical clustering of unifrac distances based on SNPs called from mapping of transcriptomic reads against the predicted coding sequences of the Cladocopium goreaui genome. Both host and symbiont samples were assigned to ecological groups based on their island of origin in order to control for similarities in expression profiles derived from shared environmental effects (e.g., similar irradiance for all colonies collected at a given reef site).

Blinding

Sample collection and data analysis were performed by separate groups in order to minimize bias.

Did the study involve field work? [x] Yes [ ] No

Field work, collection and transport

Field conditions

Relevant environmental parameters for each island/site of sampling are summarized in Table 1 of the manuscript. These include data for both conditions at the time of sampling as well as relevant historical conditions.

Location

IslandName ReefSite ColonyID HostTaxon sampling_datetimeUTC lat lon Depth_m
Isla de Las Perlas S01 C001 Pocillopora grandis (SVD4) 7/17/16 16:24 8.57953 -79.02073167 6
Isla de Las Perlas S02 C001 Pocillopora grandis (SVD4) 7/18/16 15:11 8.597766667 -79.02493193 6
Isla de Las Perlas S03 C001 N/A 7/25/16 15:32 8.6501 -79.0339 NA
Coiba S01 C001 Pocillopora grandis (SVD4) 7/20/16 15:25 7.8732 -81.7942 2
Coiba S02 C001 Pocillopora grandis (SVD4) 7/21/16 15:42 7.65115 -81.6876 2
Coiba S03 C001 Pocillopora grandis (SVD4) 7/23/16 13:40 7.2092 -81.7962 3
Malpelo S01 C011 Pocillopora grandis (SVD4) 8/5/16 14:25 3.9873 -81.5915 10
Rapa Nui S01 C001 SSH5_Pver (SVD5) 9/3/16 16:30 -27.06876528 -109.3263833 12.7
Rapa Nui S02 C001 SSH5_Pver (SVD5) 9/4/16 15:40 -27.06851667 -109.3207167 14.1
Rapa Nui S03 C001 SSH5_Pver (SVD5) 9/6/16 15:55 -27.12205 -109.428725 NA
Rapa Nui S04 C001 SSH5_Pver (SVD5) 9/7/16 15:31 -27.1478551 -109.4393007 10.6
Ducie S01 C001 Pocillopora grandis (SVD4) 9/13/16 16:40 -24.67746667 -124.7701 10
Ducie S02 C001 N/A 9/14/16 16:30 -24.69686667 -124.7944833 16.3
Ducie S03 C001 SSH5_Pver (SVD5) 9/15/16 16:30 -24.6797 -124.8018167 11.5
Gambier S01 C001 Pocillopora effusa (SVD1) 9/21/16 19:00 -23.0748 -135.0172667 11.1
Gambier S02 C001 Pocillopora effusa (SVD1) 9/23/16 17:34 -23.1651 -134.8479797 8.5
Gambier S03 C001 Pocillopora effusa (SVD1) 9/24/16 18:00 -23.23733333 -134.9566 5.2
Moorea S01 C001 SSH5_Pver (SVD5) 11/6/16 22:05 -17.48256667 -149.8864667 12
Moorea S02 C001 SSH5_Pver (SVD5) 11/7/16 19:52 -17.51843333 -149.924 12.6
Moorea S03 C001 SSH5_Pver (SVD5) 11/8/16 19:12 -17.48966667 -149.75505 10.2
Aitutaki S01 C001 Pocillopora verrucosa (SVD3) 11/14/16 0:30 -18.83996667 -159.8009 10
Aitutaki S02 C001 Pocillopora verrucosa (SVD3) 11/15/16 18:50 -18.91396667 -159.8451 12
Aitutaki S03 C001 Pocillopora verrucosa (SVD3) 11/16/16 17:54 -18.8678 -159.8186839 10.5
Niue S01 C001 Pocillopora verrucosa (SVD3) 11/22/16 19:40 -19.11343333 -169.9142333 11.5
Niue S02 C001 Pocillopora verrucosa (SVD3) 11/23/16 19:32 -18.98541667 -169.9032667 7.4
Niue S03 C001 Pocillopora meandrina (SVD2) 11/24/16 20:13 -19.04181667 -169.9185 7.9
Upolu S01 C001 Pocillopora meandrina (SVD2) 11/30/16 19:07 -14.01776667 -171.8425 11.5
Upolu S02 C001 Pocillopora meandrina (SVD2) 12/1/16 17:51 -14.06144655 -171.4303667 6.1
Upolu S03 C001 Pocillopora meandrina (SVD2) 12/2/16 18:41 -13.91876667 -171.5415667 7
Guam S01 C001 Pocillopora meandrina (SVD2) 1/28/17 23:15 13.24973333 144.64495 4.3
Guam S02 C001 Pocillopora verrucosa (SVD3) 1/29/17 22:28 13.4172 144.6446333 4.6
Guam S03 C001 Pocillopora verrucosa (SVD3) 1/30/17 23:08 13.3434 144.6361 10.2

Access & import/export

Authorization for sampling was provided under the following sampling and export permits (CITES): for PANAMA (Las Perlas) under the reference 'SEX/A-72-16' delivered by the Autoridad Nacional del Ambiente (ANAM) de la República de Panamá – Autoridad Administrativa CITES on the 28/07/2016; CITES final import permit under the reference 'FR1609100066-I' delivered the 04/08/2016 by the DRIEE ILE-DE-FRANCE; CITES export permit for PANAMA (Coiba) under the reference 'SEX/A-72-16' delivered by the Autoridad Nacional del Ambiente (ANAM) de la República de Panamá – Autoridad Administrativa CITES on the 28/07/2016; CITES final import permit under the reference 'FR1609100066-I' delivered the 04/08/2016 by the DRIEE ILE-DE-FRANCE; CITES export permit for COLOMBIA (Malpelo) under the reference '41499' delivered by the Ministerio de Ambiente y Desarrollo Sostenible de la República de Colombia on the 13/02/2017; CITES final import permit under the reference 'FR1707506158-I' delivered the 17/03/2017 by the DRIEE ILE-DE-FRANCE; CITES export permit for CHILE (Rapa Nui) under the reference '16CL000007WS' delivered by the Servicio Nacional de Pesca y Acuicultura on the 02/09/2016; CITES final import permit under the reference 'FR1607525599-I' delivered the 03/11/2016 by the DRIEE ILE-DE-FRANCE; CITES export permit for UNITED-KINGDOM (Ducie) under the reference 'FR1698700198-E' delivered by the Haut-Commissariat de la République en Polynésie Française on the 03/11/2016; CITES final import permit under the reference 'FR1607525646-I' delivered the 04/11/2016 by the DRIEE ILE-DE-FRANCE; CITES export permit for FRENCH POLYNESIA (Gambier) under the reference 'FR1698700198-E' delivered by the Haut-Commissariat de la République en Polynésie Française on the 03/11/2016; CITES final import permit under the reference 'FR1607525646-I' delivered the 04/11/2016 by the DRIEE ILE-DE-FRANCE; CITES export permit for MOOREA under the reference 'FR1698700218-E' delivered by the Haut-Commissariat de la République en Polynésie Française on the 21/11/2016; CITES final import permit under the reference 'FR1707503441-I ' delivered the 07/02/2017 by the DRIEE ILE-DE-FRANCE; CITES export permit for COOK (Aitutaki) under the reference 'CK/2016 – 14278' delivered by the Tu'anga Taporoporo national environment service of the Cook Islands on the 17/11/2016; CITES final import permit under the reference 'FR1707503442-I ' delivered the 07/02/2017 by the DRIEE ILE-DE-FRANCE; CITES export permit for NIUE under the reference 'N/A' delivered by the N/A on the N/A; CITES final import permit under the reference 'FR1707511900-I' delivered the 11/06/2017 by the DRIEE ILE-DE-FRANCE; CITES export permit for SAMOA (Upolu) under the reference 'SAMC16012' delivered by the

	Ministry of Natural Resources and Environment (MNRE) of the Government of Samoa on the 29/11/2016; CITES final import permit under the reference 'FR1707503440-I' delivered the 07/02/2017 by the DRIEE ILE-DE-FRANCE; CITES export permit for GUAM under the reference '17US18844C/9' delivered by the U.S. Fish and Wildlife service – Division of management authority – Branch of permits on the 02/03/2017; CITES final import permit under the reference 'FR1707503440-I' delivered the 07/02/2017 by the DRIEE ILE-DE-FRANCE.
Disturbance	Fragment sizes were as small as possible to minimize harm to the sampled colonies.

Reporting for specific materials, systems and methods

We require information from authors about some types of materials, experimental systems and methods used in many studies. Here, indicate whether each material, system or method listed is relevant to your study. If you are not sure if a list item applies to your research, read the appropriate section before selecting a response.

Materials & experimental systems

n/a	Involved in the study
☒	☐ Antibodies
☒	☐ Eukaryotic cell lines
☒	☐ Palaeontology and archaeology
☐	☒ Animals and other organisms
☒	☐ Human research participants
☒	☐ Clinical data
☒	☐ Dual use research of concern

Methods

n/a	Involved in the study
☒	☐ ChIP-seq
☒	☐ Flow cytometry
☒	☐ MRI-based neuroimaging

Animals and other organisms

Policy information about studies involving animals; ARRIVE guidelines recommended for reporting animal research

Laboratory animals	This study did not involve laboratory animals.
Wild animals	We intended to sample coral colonies of a single species (Pocillopora meandrina) at each island. However, later analysis revealed that five species were collected (P. meandrina, P. cf. effusa, P. verrucosa, P.grandis, and an undescribed species similar to P. verruocosa). Each colony was first photographed using a 20 cm quadrat as a scale, their depth recorded, and then sampled to collect about 70 g of tissue by mechanical fragmentation using hammer and chisel. Fragments were placed in Ziploc bags labeled by unique colony ID and brought back to the boat for further processing. All wild parent colonies remained alive at the time of sampling.
Field-collected samples	Coral fragments were lysed and homogenized in DNA/RNA shield in the field and transported at -20 C until final storage at -80 C.
Ethics oversight	No ethical approval was required as analysis were conducted on non-vertebrate species.

Note that full information on the approval of the study protocol must also be provided in the manuscript.
